# Synthesis and Characterization of Unusual *S*=1 Fe(0)‐Silyl Complexes Supported by Styrene Ligands

**DOI:** 10.1002/anie.202520739

**Published:** 2025-12-30

**Authors:** Alexis K. Bauer, Agamemnon E. Crumpton, Michael L. Neidig

**Affiliations:** ^1^ Department of Chemistry, University of Oxford Inorganic Chemistry Laboratory South Parks Road Oxford OX1 3QR UK

**Keywords:** High‐Spin, Iron(0), Low‐Valent, Mössbauer, Silicon

## Abstract

In this research we report unprecedented high‐spin (*S *= 1) Fe(0)‐silyl complexes stabilized by simple styrene ligands. Access to these novel complexes follows a straightforward route through previously reported Fe(0)‐alkyl complexes paired with a range of aryl silanes to promote the protonation of the alkyl substituent and subsequent coordination of the silicon moiety. Spectroscopic and structural analyses (SC‐XRD, Evans Method, 80 K ^57^Fe Mössbauer) established their unique electronic and structural configuration while DFT calculations further probed electronic structure and bonding effects of the styrene to Fe(0) centres, identifying essential electronic stability. By uncovering the first high‐spin (*S *= 1), alkene stabilized Fe(0)‐silyl complexes, this work offers further fundamental insights into Fe–Si bonding that can contribute to future insights into reactivity.

## Graphical abstract

Despite the long‐standing importance of iron‐silicon bonds as related to catalysis, high‐spin Fe(0)‐silyl complexes remain a historical synthetic challenge in the field. Herein, we report the first series of high‐spin *S *= 1, styrene‐stabilized Fe(0)‐silyl complexes followed by extensive characterization via Mössbauer spectroscopy, single crystal X‐ray diffraction, Evans NMR, and computational analysis. (404 characters)

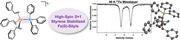

The pursuit of well‐defined iron complexes featuring Fe─Si bonds has attracted considerable attention since the early 1980s, motivated by the proposed roles of such species in iron‐catalyzed reactions including hydrosilylation, dehydrocoupling, allylic C–H silylation, transfer hydrogenation, hydroboration (where the Fe─Si bond is in the role of a pre‐catalyst), dehydrogenative borylation of olefins, and alkoxylation of silanes with alcohols.^[^ ^1^, ^2^, ^3^, ^4^, ^5^, ^6^, ^7^, ^8^, ^9^, ^10^, ^11^, ^12^, ^13^, ^14^ ^]^ Further interest was motivated by the desire to develop fundamental understanding of transition metal‐main group bonded complexes, aimed at elucidating the role of orbital interactions in relation to complex stability and reactivity.^[^ ^15^, ^16^ ^]^

Towards these goals, a variety of Fe─Si complexes have been accessed through synthetic routes including oxidative addition, salt metathesis, and σ‐bond metathesis to coordinate the silicon atom to the iron centre. The diversity of the supporting ligands on Fe has remained predominately limited to strong field ligands, resulting in a further restriction of the spin states of Fe‐silyl species. For example, CO and Cp* (1,2,3,4,5‐pentamethylcyclopentadienyl) ligands (Figure 1a) have been heavily favored in literature towards the formation of novel Fe─Si bonds, resulting in the formation of multiple examples of well‐defined Fe(II)–Si complexes all with low‐spin ground states. Such complexes are typically formed via the use of Na_2_Fe(CO)_4_ or NaFe(CO)_2_Cp* salts, though this remains an unfavorable route of formation due to the required thermal or photochemical desorption for further modification.^[^ ^17^, ^18^, ^19^, ^20^ ^]^ As a result, phosphorous‐containing pincer type complexes have been developed, including pincers containing H–Si moieties to promote Fe─Si bond formation through “chelate assisted” oxidative addition to Fe(0) via cleavage of a protonated silicon (Figure 1b).^[^ ^21^, ^22^, ^23^ ^]^ Subsequent reduction of the low‐spin Fe(II)‐silyl complexes has resulted in analogous low‐spin Fe(0)‐silyl species through CO and N_2_ coordination.^[^ ^15^, ^24^ ^]^ However, these complexes remain largely restricted for future modifications due to the overwhelming stabilizing effect of the pincer ligand. Another approach to Fe─Si bond formation has involved select examples of N‐heterocyclic silylene (NHSi) supported low‐spin Fe─Si complexes (Figure 1b), where the lone pair of the low‐oxidation state silicon(II) atom is capable of stabilizing the electron rich Fe(0) centre.^[^ ^25^, ^26^, ^27^, ^28^, ^29^, ^30^ ^]^

**Figure 1:**
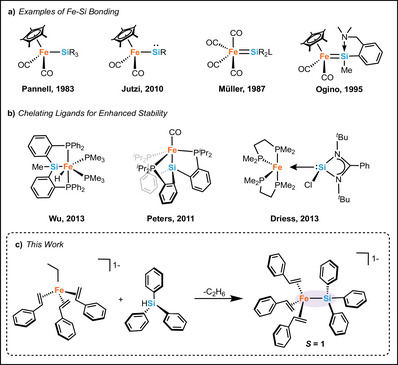
Overview of Fe─Si bonds. a) Variations of Fe─Si bonds supported by CO and Cp* ligands. b) Fe─Si bonds containing chelating phosphine ligands. c) The synthetic scheme presented in this work.

While the aforementioned examples represent an impressive array of low‐spin Fe‐silyl and related complexes, high‐spin (*S *= 1) Fe(0)‐silyl species remain fundamentally underexplored. To the best of our knowledge, the closest related example contains two η^2^–Si–H ligands, rather than the tradition silyl substituent, and is proposed to adopt an *S *= 1 ground state, though the use of a redox non‐innocent ligand creates ambiguity regarding the oxidation state of the Fe centre.^[^ ^31^ ^]^ More fundamentally, there are currently no examples in literature of Fe─Si bonds supported only by coordination of alkenes to the iron centre. This remains a stark limitation in the field due to widespread proposals of Si‐Fe‐alkene intermediates involved in reactions such as hydrosilylation.^[^ ^32^ ^]^ Moreover, the commonly proposed Chalk‐Harrod mechanism, which invokes η^2^‐coordination to the iron centre by the alkene (most often styrene type derivatives), has lacked critical structural evidence.^[^ ^10^, ^32^, ^33^, ^34^, ^35^, ^36^, ^37^, ^38^, ^39^, ^40^ ^]^ Therefore, in addition to addressing both the lack of fundamental knowledge regarding Fe─Si bond formation for high‐spin complexes, we were also intrigued to uncover a pathway towards Fe─Si bonds with alkene stabilization at iron.

Recent studies have identified a class of high‐spin Fe(0)‐NHC (*N*‐heterocyclic carbene) complexes, stabilized by simple styrene ligands.^[^ ^41^ ^]^ We therefore hypothesized that we could develop a similar synthetic strategy to form novel Fe─Si bonds, relying upon styrene ligands to stabilize the complex. In the current study, we present a general synthetic route to previously inaccessible S = 1 Fe(0)‐silyl complexes stabilized by supporting styrenes. Mössbauer spectroscopy and DFT calculations are used to characterize the species and gather fundamental insight into Fe─Si bonding, along with the role of alkene ligands.

Our group has previously reported that Fe(0)‐alkene complexes supported by styrene ligands, formed via reaction of FeCl_2_ with EtMgBr in the presence of TMEDA (N,N,N′,N′‐tetramethylethylenediamine) and excess styrene,^[^ ^42^, ^43^ ^]^ can be used as synthetic precursors to access *S *= 1 Fe(0)‐NHC complexes via ethyl protonation by NHC•HCl.^[^ ^41^ ^]^ Hence, we envisioned that reacting [Fe(0)(styrene)_3_Et]^–^ with silanes might provide a general synthetic route for the formation of Fe(0)–Si bonds, potentially resulting in high‐spin Fe(0) products analogous to the aforementioned *S *= 1 Fe(0)‐NHC complexes.^[^ ^41^ ^]^ Our initial studies focused on the reaction of isolated [Fe(styrene)_3_Et][MgBr(THF)_5_] (**1**) with triphenylsilane (Figure 2a). The reaction of **1** with 4 equivalents of triphenylsilane at −10 °C in tetrahydrofuran (THF) led to a color change of the solution from dark orange‐brown to bright orange within 5 min. Layering of the reaction mixture with hexanes at −30 °C overnight lead to the formation of room temperature stable orange crystalline blocks identified by single crystal X‐ray diffraction (SC‐XRD) as [Fe(0)(styrene)_3_SiPh_3_][MgBr(THF)_5_] (**2**) (Figure 2b).^[^ ^44^ ^]^ While Mössbauer spectroscopy indicated that 1 equiv. of triphenylsilane was sufficient for formation of the Fe─Si product, the use of excess silane was found to afford the highest isolated crystalline yield (60%). Based on the observed bond angles and bond lengths, **2** was determined to contain a distorted C_3v_ geometry (τ_4 _= 0.97) similar to the distorted configuration found in **1** (τ_4 _= 0.98). While the Fe─Si bond in **2** was expectedly elongated compared to the Fe‐C_Et_ bond length in **1** (2.384 Å and 2.069 Å respectively), the overall structure and Fe‐styrene bonding remain similar. The solid‐state, 80 K ^57^Fe Mössbauer spectrum of crystalline **2** is characterized by a major green species (95%) with isomer shift of δ = 0.41 mm s^−1^ and a quadrupole splitting of |*ΔE*_Q_| = 2.01 mm s^−1^ and a minor purple species (5%) identified by the parameters δ = 0.44 mm s^−1^ and a quadrupole splitting of |*ΔE*_Q_| = 0.62 mm s^−1^ (Figure 3a). The minor species is most likely a product of degradation due to the highly proton sensitive nature of these species. The ^57^Fe Mössbauer parameters are similar to those reported for **1** and consistent with a high‐spin Fe(0) product. The *S *= 1 ground state of **2** was confirmed using Evans method NMR (µ_eff_ = 3.5(2) µ_B_). Note that *S *= 1 Fe(styrene)_2_SIMes was characterized by µ_eff_ = 3.8(2) µ_B_, providing further evidence for the magnetic moment of **2** in support of a high‐spin, alkene‐stabilized Fe(0) complex.^[^ ^41^ ^]^ Both the lack of any resonance in the ^29^Si NMR spectrum of **2** and the observed broadness of resonances in the ^1^H NMR spectrum are further consistent with a *S *= 1, Fe(0)‐silyl species (See S4).

**Figure 2:**
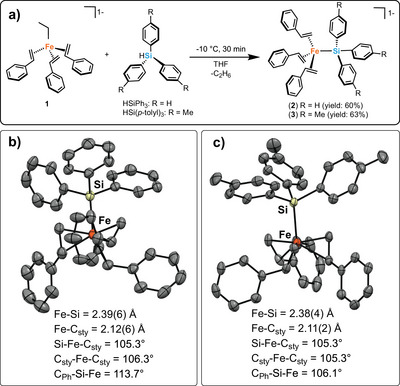
Outline of synthetic route and crystal structures for **2** and **3**. a) Synthetic route of alkene‐stabilized iron‐silyl complexes. b) Crystal structures of (B) **2**, Fe(styrene)_3_SiPh_3_, and c) **3**, Fe(styrene)_3_Si(*p‐*tolyl)_3_. The MgBr(THF)_5_ counterions and hydrogens are omitted for clarity and thermal ellipsoids are at 50% probability. All bond lengths and angles are averaged across each complex. Bond lengths and angles to *C*_sty_ are defined as the midpoint of the alkene.

**Figure 3:**
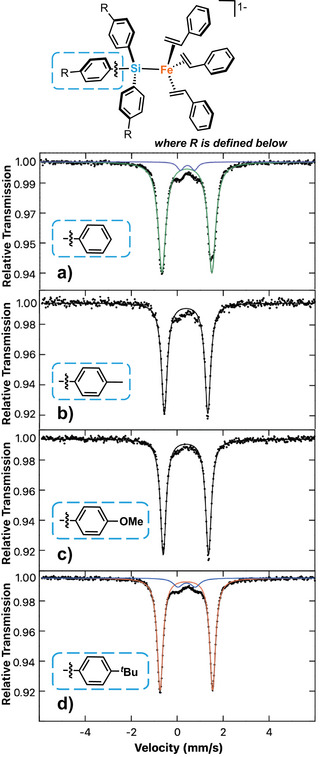
Zero‐field, 80 K ^57^Fe Mössbauer spectrum. a) complex **2** (green, 95%): δ = 0.41 mm s^−1^, |*ΔE*_Q_| = 2.01 mm s^−1^; minor species (purple, 5%): δ = 0.44 mm s^−1^, |*ΔE*_Q_| = 0.62 mm s^−1^. b) complex **3**: δ = 0.38 mm s^−1^, |*ΔE*_Q_| = 1.89 mm s^−1^. c) complex **4**: δ = 0.38 mm s^−1^, |*ΔE*_Q_| = 1.97 mm s^−1^. d) complex **5** (orange, 93%): δ = 0.40 mm s^−1^, |*ΔE*_Q_| = 2.28 mm s^−1^ minor blue (7%): δ = 0.41 mm s^−1^, |*ΔE*_Q_| = 0.76 mm s^−1^. Black dotted trace: raw data. Solid trace: best fit. Parameters for complexes also listed in Table 1.

**Table 1:** Comparison of 80 K ^57^Fe Mössbauer parameters for **1**–**5** and related complexes in literature.

Entry	Complex	Total Spin (*S*)	Isomer shift (δ) [mm s^−1^]	Quadrupole splitting (|*ΔE*_Q_|) [mm s^−1^]
1	**1** ^[^ ^43^ ^]^	1	0.37	1.37
2	**2**	1	0.41	2.01
3	**3**	1	0.38	1.89
4	**4**	1	0.38	1.97
5	**5**	1	0.40	2.28
6	Fe(styrene)_2_SIMes^[^ ^41^ ^]^	1	0.39	2.92
7	Cp*Fe(CO)_2_SiPh_3_^[^ ^18^ ^]^	0	0.04	1.80
8	(SiP*^i^* Pr_3_)FeCO^[^ ^15^ ^]^	0	0.21	1.33
9	[SiNSi]Fe(PMe_3_)_2_^[^ ^26^ ^]^	0	0.24	1.66

We next sought to expand the silanes that can be utilized in this synthetic approach to access *S *= 1 Fe(0)‐silyl complexes, focusing on alternative triaryl‐silanes. Reaction of **1** with tris(*p*‐tolyl) silane using the same synthetic procedure previously described for the synthesis of **2** resulted in an orange powder precipitating from the solution within 15 min of reacting at −10 °C, followed by filtering and washing with hexanes. **3** was subsequently obtained in crystalline form by reducing the concentration of the solution, followed by the layering of both THF and hexanes and storage at −30 °C overnight to produce bright orange crystals (63% yield) identified by SC‐XRD as [Fe(0)(styrene)_3_Si(*p*‐tolyl)_3_] [MgBr(THF)_5_] (τ_4_ = 0.98) (**3**) (Figure 2c).^[^ ^45^ ^]^ The 80 K ^57^Fe Mössbauer spectrum of **3** is characterized by parameters of δ = 0.38 mm s^−1^ and |*ΔE*_Q_| = 1.89 mm s^−1^, in good agreement with those observed previously for **2** (Figure 3b). The *S *= 1 ground state of **3** was confirmed by Evans method NMR (µ_eff_ = 3.9(2) µ_B_). Tris(4‐methoxyphenyl) and tris(4‐tertbutylphenyl) silane analogues could also be synthesized using the same general reaction of **1** with the corresponding aryl silanes (**4** and **5**, respectively), demonstrating the broader use of this synthetic procedure across a range of aryl silanes. While **4** and **5** could not be crystallized despite extensive efforts, they could be isolated as pure complexes based on Mössbauer spectroscopy (yields between 72% and 62% respectively) with Mössbauer parameters similar to **2** and **3. 4** has parameters of δ = 0.38 mm s^−1^ and |*ΔE*_Q_| = 1.97 mm s^−1^ while **5** was identified as the major species (orange, 93%) has parameters of δ = 0.40 mm s^−1^ and quadrupole splitting of |*ΔE*_Q_| = 2.28 mm s^−1^ with a minor degradation species (blue, 5%, δ = 0.41 mm s^−1^ and |*ΔE*_Q_| = 0.76 mm s^−1^) (Figure 3c and d). The similar isomer shifts across **2**–**5** is consistent with similar Fe─Si bonding across this series of the silanes. Synthesis attempts with non‐aryl silanes resulted in Mössbauer spectra containing several unidentifiable species, indicating the use of aryl silanes may provide required steric stability to isolate the high‐spin Fe(0)‐silyl complexes (Table 1).

Despite evaluation under various conditions, the isolated complexes exhibited no discernible reactivity as a pre‐catalyst for hydrosilylation, however, the ability to access S = 1 Fe(0)‐silyls provides a useful platform for comparison with previously published low‐spin Fe(0)‐silicon complexes. The Fe─Si bond lengths (e.g., 2.39(6) and 2.38(4) Å for **2** and **3**, respectively) are on the higher end of the crystallographically published typical range for single Fe─Si bonds throughout literature (2.20–2.35 Å). ^[^ ^17^, ^24^, ^33^, ^44^, ^46^ ^]^ For example, Pannell and coworkers reported a Fe─Si bond length of 2.34 Å in Fe(CO)_2_Cp(SiMe_2_SiPh_3_), with the silyl group acting as a σ‐donor towards the Fe.^[^ ^17^ ^]^ Moreover, Fe─Si complexes containing pincer ligands, such as those produced by Peters and coworkers, contain Fe‐Si bond lengths of approximately 2.25–2.30 Å, with the shortest example highlighting the back bonding from the Fe centre to silicon. ^[^ ^15^, ^24^, ^44^, ^46^, ^47^ ^]^ A more obvious difference lies in the comparison of Mössbauer parameters, particularly the isomer shifts as a reflection of *s*‐electron density around the Fe centre. Of the alkene‐stabilized Fe(0) complexes (entries 1–6), the isomer shift remains largely the same (0.37–0.41 mm s^−1^) while maintaining the same oxidation and spin state with either Fe‐C_Et_ or Fe‐Si bonds. A Mössbauer spectrum comparison with the four‐coordinated Cp*Fe(CO)_2_SiPh_3_ exhibits the effect of strong field ligands on the Fe nucleus with an isomer shift of δ = 0.04 mm s^−1^ (entry 7).^[^ ^17^ ^]^ Similarly, a Mössbauer spectrum published by Peters and coworkers involving their (SiP*^i^* Pr_3_)FeCO pincer complex shows an isomer shift of δ = 0.21 mm s^−1^, owing to the low‐spin state of the complex (entry 8).^[^ ^15^ ^]^ The [SiNSi] pincer variant (SiNSi = bis(silylene) pyridine) published by Driess and coworkers follows in a similar trend to Peters and coworkers (δ = 0.24 mm s^−1^), further establishing a precedent of Mössbauer parameters for low‐spin Fe─Si pincer complexes (entry 9).^[^ ^26^ ^]^ Overall, the isomer shift of low‐spin Fe(0) is markedly lower than that of the high‐spin species, forming a distinct characteristic, and developing a foundation for characterizing future high‐spin Fe(0) silyls.

Computational analysis was employed to gain further insight into electronic structure and bonding in this unusual class of *S *= 1 Fe(0)–Si bonded complexes. To begin, the geometries of complexes **1–5** were optimized using TPSSh/x2c‐TZVPPall and the calculated bond lengths and angles were found to be in good agreement with those crystallographically determined. For **2**, the Fe─Si bond was calculated as 2.41 Å (2.39 Å experimental) and the C_Ph_‐Si‐Fe bond angle 115.25° (113.7° experimental). Further support for the quality of the computational models originates from the calculated Mössbauer parameters for **2**, which gave good agreement with experiment (wb97x/x2c‐TZVPPall with iron at CP(PPP): δ = 0.38 mm s^−1^ and |*ΔE*_Q_| = 1.97 mm s^−1^ (calc) versus δ = 0.41 mm s^−1^ and |*ΔE*_Q_| = 2.01 mm s^−1^ (exp)). Similar correspondence of calculated versus experimental Mossbauer parameters were also found for complexes **3**, **4**, and **5** as expected due to their similar structures (see Supporting Information), as well as complex **1** (δ = 0.35 mm s^−1^ and |*ΔE*_Q_| = 1.18 mm s^−1^) and Fe(styrene)_2_SIMes (δ = 0.33 mm s^−1^ and |*ΔE*_Q_| = 2.48 mm s^−1^).

We then determined the Mayer natural bond order to compare the Fe‐C_Et_ and Fe─Si bonding in complexes **1** and **2**, respectively. ^[^ ^48^, ^49^ ^]^ The Fe─Si bond order of 0.66 calculated for **2** indicates that the Fe‐Si bond is stronger that the analogous Fe‐C bond in **1** (0.45) and Fe(styrene)_2_SIMes (0.50) (See SI22 for computational details). This trend is consistent with a more electropositive silicon as compared to carbon, and therefore the silicon is able to engage in stronger sigma donation to the Fe centre. In contrast, the Fe‐styrene bond order is quite weak for **1** (0.44) and therefore remains similar for both **2** (0.42) and Fe(styrene)_2_SIMes (0.46). It is interesting to note that complexes **1–5** adopt a distorted C_3v_ geometry, and DFT was utilized to further examine the bonding and electronic structure in these complexes. Analysis of the β ‐spin orbital contributions for **1** and **2** reveals a notable difference in the amount of d‐character present in their unoccupied d‐type molecular orbitals; **2** shows significantly greater d‐character (42% and 47%) than **1** (29% and 32%) (Figure 4). The significant difference in d‐character for the unoccupied antibonding orbitals is consistent with the higher Mayer bond order of **2** for the Fe─Si interaction, reflecting stronger σ‐donation from silicon. The distorted C_3v_ environment promotes mixing among the metal centred orbitals, consistent with the DFT derived orbital compositions. Therefore, the reported values representing the total d‐orbital contribution to each active molecular orbital and a complete analysis, including the corresponding alpha‐spin contributions can be found in the Supporting Information. Overall, the DFT analysis supports a framework in which silicon donates more strongly to the iron centre than carbon, resulting in an increased Fe─Si bond order. Note that similar calculations were also performed for Fe(styrene)_2_SIMes for comparison to the Fe─Si and Fe‐C_Et_ moieties (see Supporting Information) in order to document the unique bonding nature of high‐spin Fe(0) complexes.

**Figure 4:**
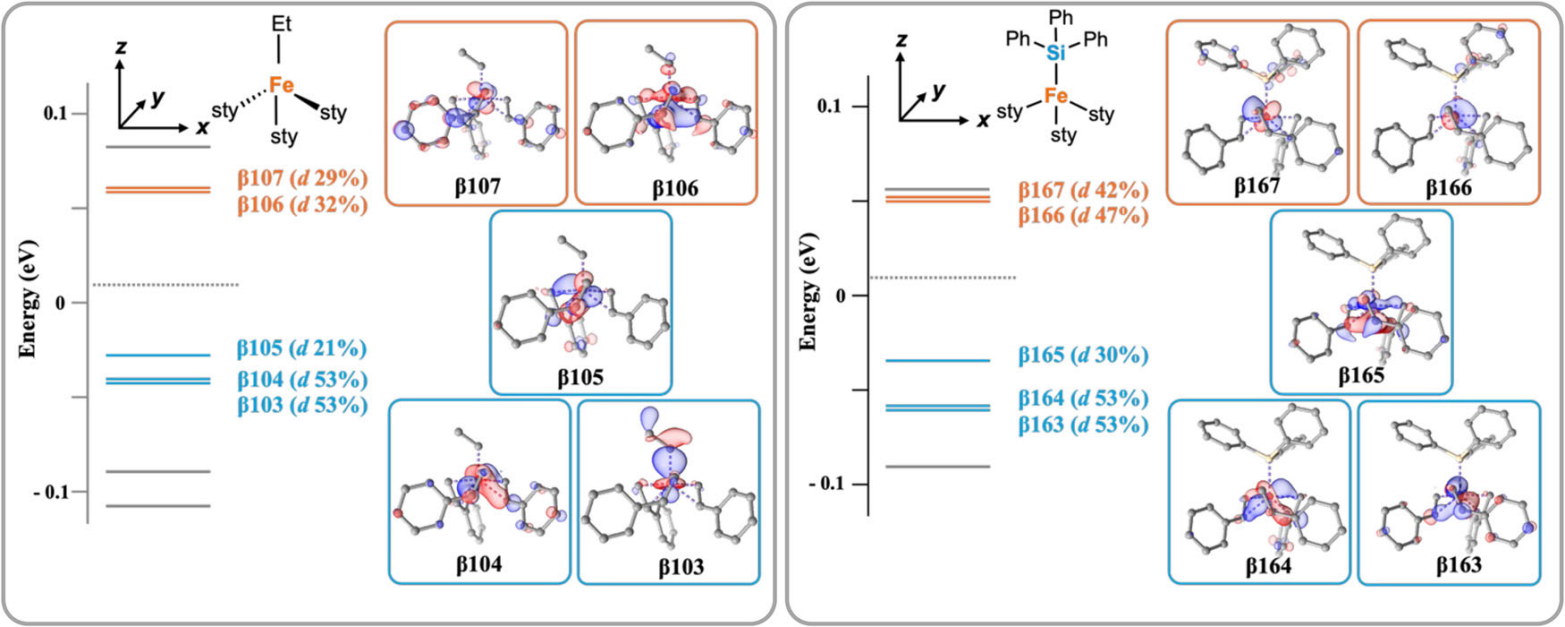
Calculated molecular orbitals (β) for Fe(styrene)_3_Et (1, left) and Fe(styrene)_3_SiPh_3_ (2, right). Percentages of d‐character (%) represent a summation of d‐character of the molecular orbital. Reported values and complete analysis of d‐orbital contribution can be found in the SI. The molecular orbitals in blue represent occupied orbitals whereas orange represents unoccupied orbitals. The dashed line exhibits the division between the occupied and unoccupied states.

We have disclosed the first example of low‐valent, high‐spin (*S* = 1) Fe(0)‐silyl complexes stabilized by styrene ligands with a variety of aryl silanes. Extensive spectroscopic, structural and computational analyses provide insights into their unique bonding and electronic structures. This work establishes an efficient and simplified synthetic pathway for the generation of novel high‐spin Fe(0)‐silyl complexes, laying the foundation for further investigation of synthetic pathways towards the formation of Fe─Si bonds. Overall, the generation of unique high‐spin Fe(0)‐silyl complexes expands the understanding of Fe‐Si bond formations and provides opportunities for future ventures in synthesis and reactivity Fe‐Si chemistry.

## Supporting Information

The authors have cited additional references within the Supporting Information.^[50–80]^

## Conflict of Interests

The authors declare no conflict of interest.

## Supporting information



Supporting Information

Supporting Information

## Data Availability

The data that support the findings of this study are available in the supplementary material of this article.

## References

[1] C. V. Thompson , H. D. Arman , Z. J. Tonzetich , Organometallics 2022, 41, 430–440, 10.1021/acs.organomet.1c00682.

[2] A. M. Tondreau , C. C. H. Atienza , K. J. Weller , S. A. Nye , K. M. Lewis , J. G. P. Delis , P. J. Chirik , Science 2012, 335, 567–570, 10.1126/science.1214451. 22301315

[3] J. V. Obligacion , P. J. Chirik , Nat. Rev. Chem. 2018, 2, 15–34, 10.1038/s41570-018-0001-2. 30740530 PMC6365001

[4] X. Du , Z. Huang , ACS Catal. 2017, 7, 1227–1243, 10.1021/acscatal.6b02990.

[5] M. Zhang , A. Zhang , Appl. Organomet. Chem. 2010, 24, 751–757, 10.1002/aoc.1701.

[6] M. A. Farcaş‐Johnson , D. Gasperini , A. K. King , S. Mohan , A. N. Barrett , S. Lau , M. F. Mahon , Y. Sarazin , S. H. Kyne , R. L. Webster , Organometallics 2023, 42, 3013–3024, 10.1021/acs.organomet.3c00339. 37886624 PMC10598884

[7] P. Zhang , X. Li , X. Qi , H. Sun , O. Fuhr , D. Fenske , RSC Adv. 2018, 8, 14092–14099, 10.1039/C8RA02606H. 35539322 PMC9079873

[8] Z. Guo , H. Wen , G. Liu , Z. Huang , Org. Lett. 2021, 23, 2375–2379, 10.1021/acs.orglett.1c00670. 33689387

[9] Á. Raya‐Barón , P. Oña‐Burgos , I. Fernández , ACS Catal. 2019, 9, 5400–5417, 10.1021/acscatal.9b00201.

[10] B. Marciniec , A. Kownacka , I. Kownacki , R. Taylor , Appl. Catal., A 2014, 486, 230–238, 10.1016/j.apcata.2014.08.037.

[11] C.‐H. Guo , X. Liu , J. Jia , H.‐S. Wu , Computat. Theor. Chem. 2015, 1069, 66–76, 10.1016/j.comptc.2015.07.007.

[12] P. He , M.‐H. Guan , M.‐Y. Hu , Y.‐J. Zhou , M.‐Y. Huang , S.‐F. Zhu , Angew. Chem. Int. Ed. 2024, 63, e202402044. 10.1002/anie.202402044 38469657

[13] A. Sen , R. Kumar , T. Tewari , R. G. Gonnade , S. H. Chikkali , Chem. ‐ Eur. J. 2023, 29, e202301375, 10.1002/chem.202301375. 37285327

[14] N. Ishiwata , T. Komuro , K. Takahashi , Y. Zenzai , H. Tobita , H. Hashimoto , Chem. Asian. J. 2025, 20, e00709, 10.1002/asia.202500709. 40689858 PMC12392704

[15] Y. Lee , J. C. Peters , J. Am. Chem. Soc. 2011, 133, 4438–4446, 10.1021/ja109678y. 21375250

[16] L. P. Griffin , A. K. Bauer , A. E. Crumpton , M. A. Ellwanger , A. Heilmann , A. Wiesner , M. L. Neidig , S. Aldridge , Chem. ‐ Eur. J. 2025, 31, e202404451, 10.1002/chem.202404451. 39960353 PMC11937870

[17] L. Párkányi , K. H. Pannell , C. Hernandez , J. Organomet. Chem. 1983, 252, 127–132, 10.1016/0022-328X(83)80075-8.

[18] K. H. Pannell , C. C. Wu , G. J. Long , J. Organomet. Chem. 1980, 186, 85–90, 10.1016/S0022-328X(00)93820-8.

[19] P. Jutzi , K. Leszczyńska , A. Mix , B. Neumann , B. Rummel , W. Schoeller , H.‐G. Stammler , Organomeallics 2010, 29, 4759–4761.

[20] C. Zybill , G. Müller , Angew. Chem. Int. Ed. Eng. 1987, 26, 669–670, 10.1002/anie.198706691.

[21] S. Wu , X. Li , Z. Xiong , W. Xu , Y. Lu , H. Sun , Organometallics 2013, 32, 3227–3237, 10.1021/om400047j.

[22] C. V. Thompson , H. D. Arman , Z. J. Tonzetich , Organometallics 2019, 38, 2979–2989, 10.1021/acs.organomet.9b00335.

[23] Z. J. Tonzetich , L. H. Do , S. J. Lippard , J. Am. Chem. Soc. 2009, 131, 7964–7965, 10.1021/ja9030159. 19459625 PMC2713435

[24] N. P. Mankad , M. T. Whited , J. C. Peters , Angew. Chem. Int. Ed. 2007, 46, 5768–5771, 10.1002/anie.200701188. 17600810

[25] B. Blom , S. Enthaler , S. Inoue , E. Irran , M. Driess , J. Am. Chem. Soc. 2013, 135, 6703–6713, 10.1021/ja402480v. 23570308

[26] D. Gallego , S. Inoue , B. Blom , M. Driess , Organometallics 2014, 33, 6885–6897, 10.1021/om500966t.

[27] M. M. Hänninen , K. Pal , B. M. Day , T. Pugh , R. A. Layfield , Dalton Trans. 2016, 45, 11301–11305, 10.1039/C6DT02486F. 27362948

[28] L. Witteman , M. Lutz , M.‐E. Moret , Organometallics 2018, 37, 3024–3034, 10.1021/acs.organomet.8b00399. 30270963 PMC6158677

[29] X. Qi , T. Zheng , J. Zhou , Y. Dong , X. Zuo , X. Li , H. Sun , O. Fuhr , D. Fenske , Organometallics 2019, 38, 268–277, 10.1021/acs.organomet.8b00700.

[30] M. J. Krahfuß , J. Nitsch , F. M. Bickelhaupt , T. B. Marder , U. Radius , Chem. ‐ Eur. J. 2020, 26, 11276–11292. 32233000 10.1002/chem.202001062 PMC7497151

[31] S. C. Bart , E. Lobkovsky , P. J. Chirik , J. Am. Chem. Soc. 2004, 126, 13794–13807, 10.1021/ja046753t. 15493939

[32] M. D. Greenhalgh , A. S. Jones , S. P. Thomas , ChemCatChem 2015, 7, 190–222, 10.1002/cctc.201402693.

[33] D. C. Najera , M. N. Peñas‐Defrutos , M. García‐Melchor , A. R. Fout , Inorg. Chem. 2024, 63, 17706–17713, 10.1021/acs.inorgchem.4c02533. 39254604 PMC11423403

[34] J. Guo , Z. Cheng , J. Chen , X. Chen , Z. Lu , Acc. Chem. Res. 2021, 54, 2701–2716, 10.1021/acs.accounts.1c00212. 34011145

[35] M.‐Y. Hu , P. He , T.‐Z. Qiao , W. Sun , W.‐T. Li , J. Lian , J.‐H. Li , S.‐F. Zhu , J. Am. Chem. Soc. 2020, 142, 16894–16902, 10.1021/jacs.0c09083. 32945664

[36] Y. Sunada , D. Noda , H. Soejima , H. Tsutsumi , H. Nagashima , Organometallics 2015, 34, 2896–2906, 10.1021/acs.organomet.5b00201.

[37] Y. Maruyama , K. Yamamura , I. Nakayama , K. Yoshiuchi , F. Ozawa , J. Am. Chem. Soc. 1998, 120, 1421–1429, 10.1021/ja973718w.

[38] F. Seitz , M. S. Wrighton , Angew. Chem. Int. Ed. Eng. 1988, 27, 289–291, 10.1002/anie.198802891.

[39] A. Sen , R. Kumar , T. Tewari , R. Kumar , C. P. Vinod , H. Sharma , K. Vanka , S. H. Chikkali , Catal. Sci. Technol. 2024,14, 2752.

[40] N. G. Simonian , M. Feo , C. Tanguy , C. Troufflard , G. Lefevre , ACS Cat. 2024, 14 12163–12172.

[41] Z.‐J. Zhang , N. Jacob , S. Bhatia , P. Boos , X. Chen , J. C. DeMuth , A. M. Messinis , B. B. Jei , J. C. A. Oliveira , A. Radović , M. L. Neidig , J. Wencel‐Delord , L. Ackermann , Nat. Commun. 2024, 15, 3503. 38664372 10.1038/s41467-024-47589-7 PMC11045758

[42] P. G. N. Neate , M. D. Greenhalgh , W. W. Brennessel , S. P. Thomas , M. L. Neidig , J. Am. Chem. Soc. 2019, 141, 10099–10108, 10.1021/jacs.9b04869. 31150210 PMC6686191

[43] P. G. N. Neate , M. D. Greenhalgh , W. W. Brennessel , S. P. Thomas , M. L. Neidig , Angew. Chem. Int. Ed. 2020, 59, 17070–17076, 10.1002/anie.202006639. PMC7895456 32542848

[44] R. Imayoshi , K. Nakajima , J. Takaya , N. Iwasawa , Y. Nishibayashi , Eur. J. Inorg. Chem. 2017, 2017, 3769–3778.

[45] Deposition numbers 2484414 (for **2**) and 2484415 (for **3**) contain the supplementary crystallographic data for this paper. These data are provided free of charge by the joint Cambridge Crystallographic Data Centre and Fachinformationszentrum Karlsruhe.

[46] L. J. Murphy , M. J. Ferguson , R. McDonald , M. D. Lumsden , L. Turculet , Organometallics 2018, 37, 4814–4826, 10.1021/acs.organomet.8b00807.

[47] M. T. Whited , N. P. Mankad , Y. Lee , P. F. Oblad , J. C. Peters , Inorg. Chem. 2009, 48, 2507–2517, 10.1021/ic801855y. 19209938

[48] J. L. Kneebone , V. E. Fleischauer , S. L. Daifuku , A. A. Shaps , J. M. Bailey , T. E. Iannuzzi , M. L. Neidig , Inorg. Chem. 2016, 55, 272–282. 26654097 10.1021/acs.inorgchem.5b02263 PMC4887941

[49] H.‐C. Chang , B. Mondal , H. Fang , F. Neese , E. Bill , S. Ye , J. Am. Chem. Soc. 2019, 141, 2421–2434, 10.1021/jacs.8b11429. 30620571 PMC6728100

