# Supplementary material for: Synthesis and Characterization of Unusual S=1 Fe(0)‐Silyl Complexes Supported by Styrene Ligands
Source: Angew Chem Int Ed Engl. 2025 Dec 30;65(8):e20739. doi: 10.1002/anie.202520739 (PMC12910147; doi:10.1002/anie.202520739)
Supplement: Supplementary file 1 — Supporting Information [file ANIE-65-e20739-s001.pdf]

Synthesis and Characterization of Unusual *S*=1 Fe(0)-Silyl Complexes Supported by Styrene Ligands

Alexis K. Bauer, Agamemnon E. Crumpton, and Michael L. Neidig*

Department of Chemistry, University of Oxford, Oxford OX1 3QR, United Kingdom

1. EXPERIMENTAL 2

1.1 General Considerations 2

1.2 Experimental Methods 2
1.2.1 Mössbauer Spectroscopy. 2
1.2.2 ^{1}H and ^{29}Si NMR Spectroscopy. 2
1.2.3 Magnetic Susceptibility Evans Method 2
1.2.4 UV-vis Spectroscopy. 2

1.3 Preparation of Fe(0)(styrene)$_3$SiPh$_3$ (2). 2

1.4 Preparation of Fe(0)(styrene)$_3$Si(*p*-tolyl)$_3$ (3). 3

1.5 Preparation of Fe(0)(styrene)$_3$Si(4-methoxyphenyl)$_3$ (4). 3

1.6 Preparation of Fe(0)(styrene)$_3$Si(4-tert-butylphenyl)$_3$ (5 3

2. SUPPLEMENTARY SPECTROSCOPIC DATA 4

2.1 Supplementary NMR 4

2.2 UV-vis Spectroscopy Data 10

3. COMPUTATIONAL ANALYSIS 15

3.1 Computational details 15
3.1.1 Fe(Styrene)$_3$Et 16
3.1.2 Fe(Styrene)$_3$SiPh$_3$ 17
3.1.2 Fe(Styrene)$_3$Si(*p*-tolyl)$_3$ 18
3.1.2 Fe(Styrene)$_3$Si(4-methoxyphenyl)$_3$ 19
3.1.2 Fe(Styrene)$_3$Si(4-tert-butylphenyl)$_3$ 20
3.1.3 Fe(Styrene)$_2$SIMes 21
3.1.4 Mayer Bond Orders 22
3.1.5 Calculated IR Stretches 22
3.1.6 Simulated UV-vis Spectra 22

4. X-RAY CRYSTALLOGRAPHIC DATA 25

5. REFERENCES 72

1. Experimental

1.1 General Considerations

Starting material was prepared using literature protocol.[50],[51] Triphenylsilane was purchased commercially and used without additional purification. All other silanes were synthesized through the literature procedure published for tris(4-methoxyphenyl)silane using the respective aryl bromide.[2] Anhydrous THF was further dried through activated-alumina filtration and stored over 4 Å molecular sieves under an inert nitrogen atmosphere. All moisture and air-sensitive experiments were conducted in an MBraun inert-atmosphere N_2 glovebox. Additional experimental details are outlined in the electronic supporting information.

1.2 Experimental Methods

1.2.1 Mössbauer Spectroscopy. The solid Mössbauer sample was prepared in an inert atmosphere glovebox equipped with a liquid nitrogen fill port to enable sample freezing to 77 K within the glovebox. The sample was loaded into a Delrin Mössbauer measurements were performed using a See Co. MS4 Mössbauer spectrometer integrated with a Janis SVT-400T He/N2 cryostat for measurements at 80 K. Isomer shifts were determined relative to α-Fe at 298K. The Mössbauer spectrum was fit using the program WMoss (SeeCo). Errors of the fit analysis were the following: $\delta \pm 0.02$ mm/s and $\Delta E_Q \pm 3\%$.

1.2.2 ^{1}H and ^{29}Si NMR Spectroscopy. All NMR spectroscopic measurements were performed on a Bruker AVIII 500 MHz NMR spectrometer at ambient temperature. Chemical shifts (δ) are reported in parts per million (ppm) and calibrated using deuterated solvent signals for ^{1}H NMR (THF-*d*8 1.73/3.58 ppm). ^{29}Si NMR used only 1 equiv. of triphenylsilane in solution to track Fe-Si coordination.

1.2.3 Magnetic Susceptibility Evans Method. Crystalline material was isolated as reported herein, charged in a scintillation vial with a stir bar and 1.8mL of THF to obtain a concentration of 10 mM. Trifluorotoluene was added (0.2mL) to the solution and allowed to dissolve until completely dissolved. Of the solution, 0.6mL was then placed in a J. Young NMR tube. A capillary of trifluorotoluene/deuterated THF (1:1) was then placed into the tube, the tube was sealed, and frozen in liquid nitrogen until run at room temperature. Measurements were performed on a Bruker AVIIIHD 400 Nanobay. Calculations were performed in accordance with literature protocol.[52],[53]

1.2.4 UV-vis Spectroscopy. UV-vis samples were prepared at 10 μM under an inert atmosphere of N_2 and loaded into J. Young Capped UV-vis quartz cells of 1 cm path length. UV-vis spectra were recorded on a V-770 UV-Visible/NIR Spectrometer equipped with a Peltier temperature controller.

1.3 Preparation of Fe(0)(styrene)$_3$SiPh$_3$ (2). Under an inert atmosphere of N_2, Fe(styrene)$_3$Et (40 mg, 0.05 mmol), triphenylsilane (48.3 mg, 4.0 equiv., 0.20 mmol) and 4mL anhydrous tetrahydrofuran (THF) were charged to a prechilled scintillation vial equipped with a stir bar at -

10 ºC and stirred 30 minutes. The solution colour changed from a deep orange/brown to a bright orange. The solution was then layered with 2mL of hexanes and store at -30 °C overnight to result in bright orange needles. The crystalline material was filtered and washed with hexanes and dried in vacuo. (isolated yield: 30.4 mg, 60%). Anal. Calcd. [%]: C, 63.80; H, 8.20. Found: C, 62.85; H, 5.90. Magnetic moment identified as μ_{eff} = 3.5(2) μ_B. UV-vis absorption bands (1/cm): 44,385; 43, 591; 47,505. Note, **Fe(0)(styrene)$_3$Ethyl** identified with UV-vis absorption bands (1/cm): 40,322; 40,933; 46,360; 47,326.

1.4 Preparation of Fe(0)(styrene)$_3$Si(*p*-tolyl)$_3$ (3). Under an inert atmosphere of N_2, Fe(styrene)$_3$Et (40 mg, 0.05 mmol), tri-*p*-tolylsilane (28.1 mg, 2.0 equiv., 0.10 mmol) and 2mL anhydrous tetrahydrofuran (THF) were charged to a prechilled scintillation vial equipped with a stir bar at -10 ºC and stirred 15 minutes. The solution colour changed from a deep orange/brown to a bright orange, and bright orange colored powder precipitated. The solid was filtered over a frit and washed with hexanes (yield: 33.2 mg, 63%). Crystals were formed by charging Fe(styrene)$_3$Et (20 mg, 0.02 mmol), tris-*p*-tolylsilane (14.0 mg, 2.0 equiv., 0.05 mmol) and 4mL anhydrous tetrahydrofuran (THF) to a prechilled scintillation vial equipped with a stir bar at -10 ºC and stirred 15 minutes. A 0.5mL aliquot was then charged into a new, chilled scintillation vial, followed by the addition of 0.2mL chilled THF and careful layering of 0.4mL chilled hexanes. The solution was then stored at -30 °C for 2-4 days to form dark orange crystals. Anal. Calcd. [%]: C, 68.75; H, 7.41. Found: C, 68.53; H, 7.02. Magnetic moment identified as μ_{eff} = 3.9(2) μ_B. UV-vis absorption bands (1/cm): 38,834; 40,160; 43,196; 47,505.

1.5 Preparation of Fe(0)(styrene)$_3$Si(4-methoxyphenyl)$_3$ (4). Under an inert atmosphere of N_2, Fe(styrene)$_3$Et (40 mg, 0.05 mmol), tris(4-methoxyphenyl)silane (32.5 mg, 2.0 equiv., 0.10 mmol) and 2mL anhydrous tetrahydrofuran (THF) were charged to a prechilled scintillation vial equipped with a stir bar at -10 ºC and stirred 15 minutes. The solution colour changed from a deep orange/brown to a bright orange, and bronze colored powder precipitated. The solid was filtered over a frit and washed with hexanes. (yield: 39.6, 72%). Anal. Calcd. [%]: C, 62.80; H, 7.10. Found: C, 61.12; H, 6.28. UV-vis absorption bands (1/cm): 38,461; 40,160; 43,103; 47,619.

1.6 Preparation of Fe(0)(styrene)$_3$Si(4-tert-butylphenyl)$_3$ (5). Under an inert atmosphere of N_2, Fe(styrene)$_3$Et (40 mg, 0.05 mmol), tris(4-*tert*-butylphenyl)silane (39.7 mg, 2.0 equiv., 0.10 mmol) and 2mL anhydrous tetrahydrofuran (THF) were charged to a prechilled scintillation vial equipped with a stir bar at -10 ºC and stirred 15 minutes. The solution colour changed from a deep orange/brown to a bright orange, and bronze colored powder precipitated. The solid was filtered over a frit and washed with hexanes. (yield: 34.5, 62%). Anal. Calcd. [%]: C, 70.50; H, 8.21. Found: C, 70.00; H, 7.60. UV-vis absorption bands (1/cm): 38,080; 40,192; 42,918; 47,438.

2. Supplementary Spectroscopic Data

2.1 Supplementary NMR

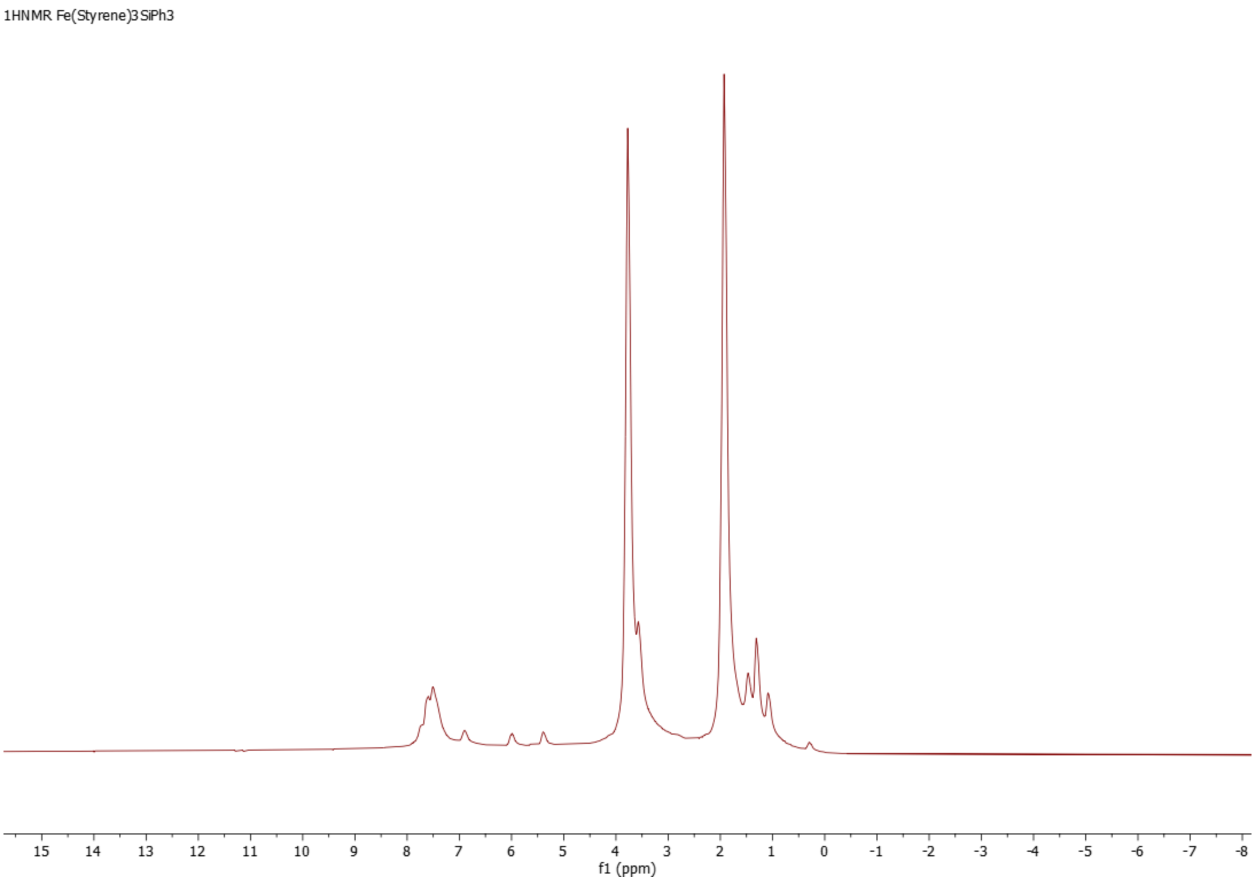

Figure S1. ^{1}H NMR spectrum of $Fe(styrene)_3SiPh_3$ in THF-d8. The broadness of the signals is consistent with the assignment of this species as paramagnetic.

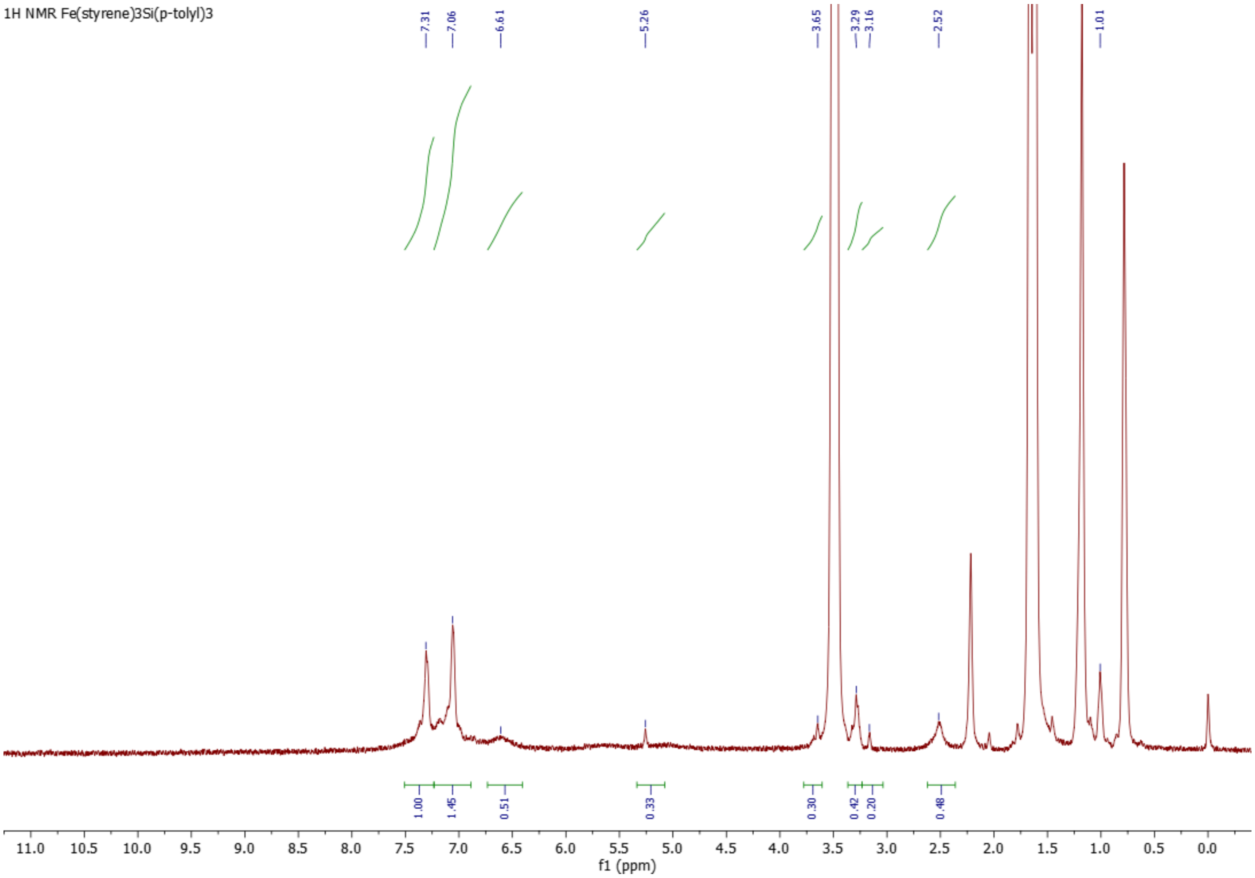

Figure S2. ^{1}H NMR spectrum of Fe(styrene)$_3$Si(tolyl)$_3$ in THF-d8. The broadness of the signals is consistent with the assignment of this species as paramagnetic. Weak signals reflect low solubility of the complex.

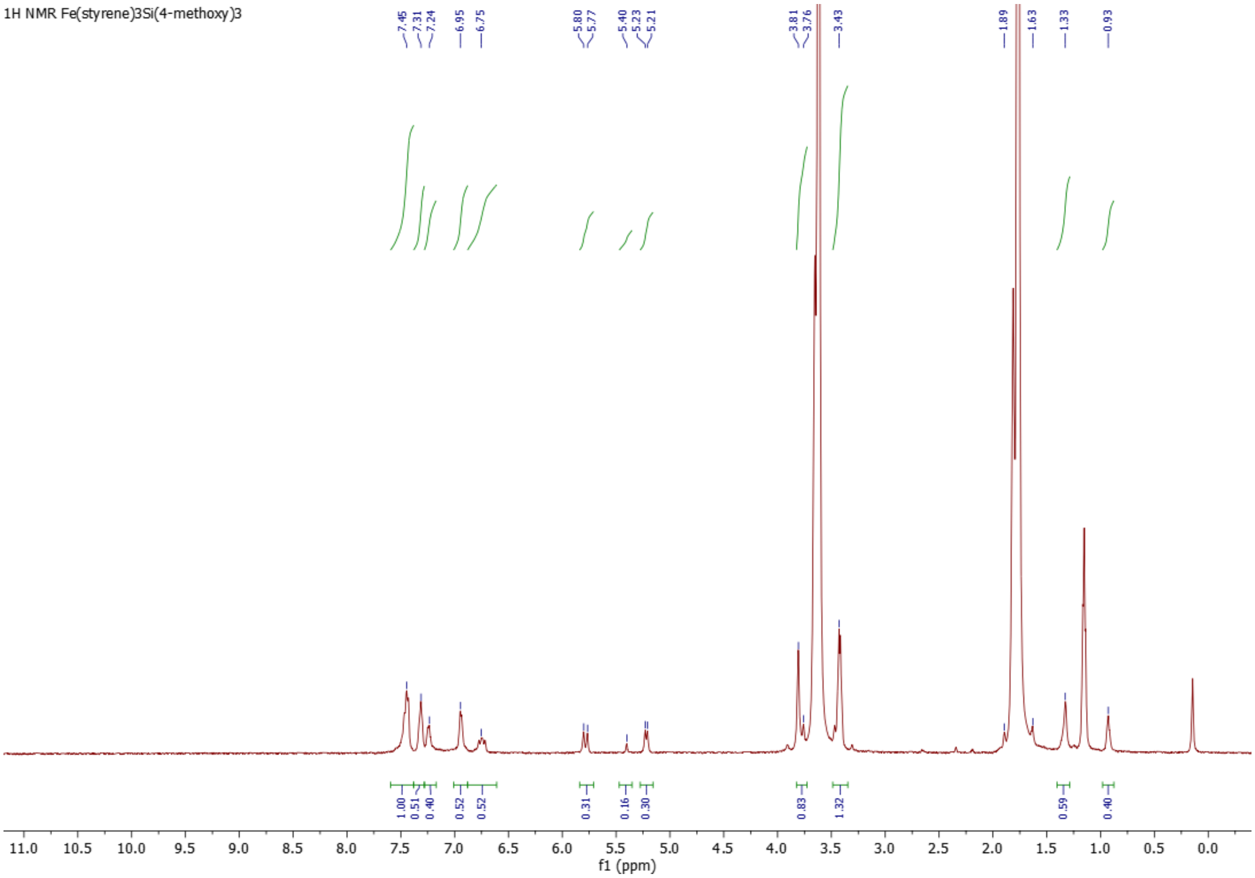

Figure S3. ^{1}H NMR spectrum of $Fe(styrene)_3Si(4\text{-methoxyphenyl})_3$ in THF-d8. The broadness of the signals is consistent with the assignment of this species as paramagnetic. Weak signals reflect low solubility of the complex.

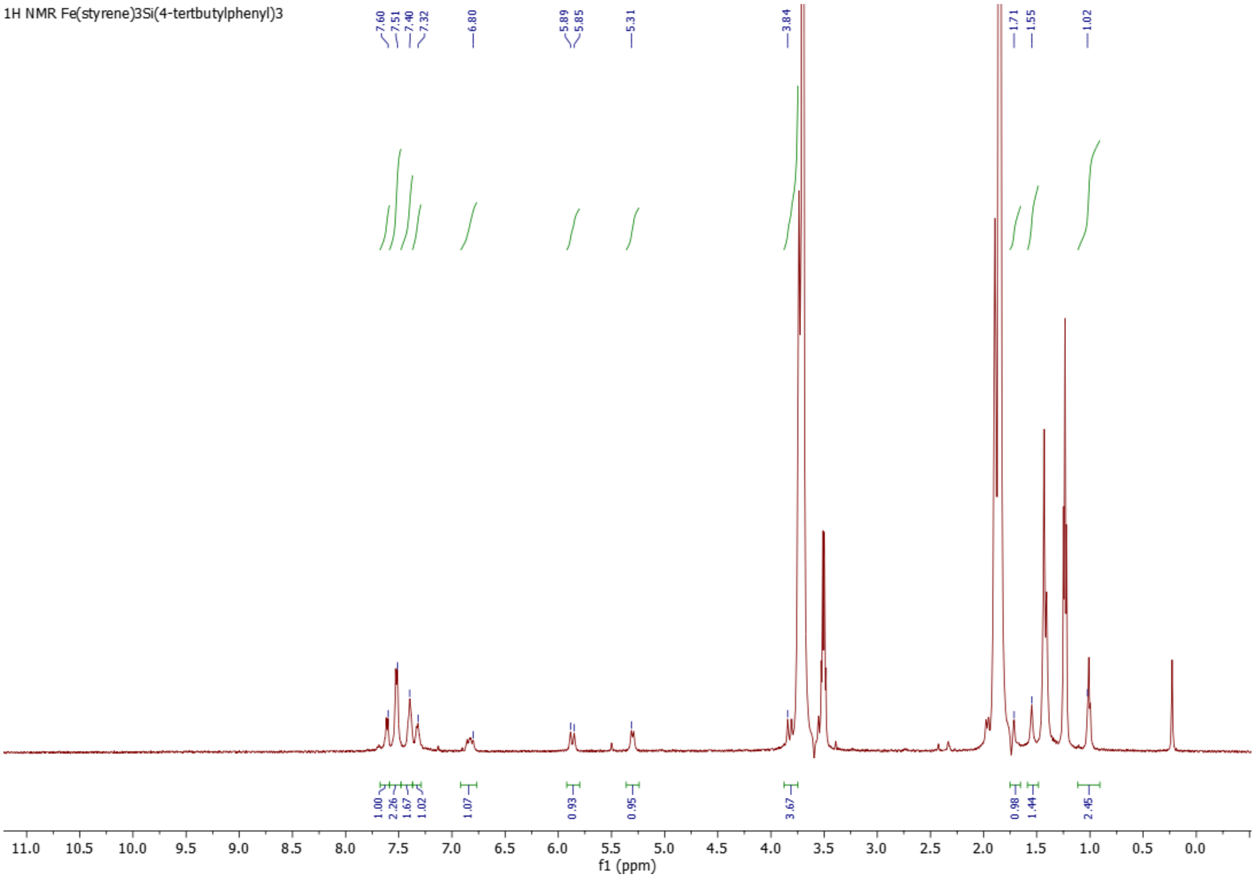

Figure S4. ^{1}H NMR spectrum of Fe(styrene)$_3$Si(4-tertbutylphenyl)$_3$ in THF-d8. The broadness of the signals is consistent with the assignment of this species as paramagnetic. Weak signals reflect low solubility of the complex.

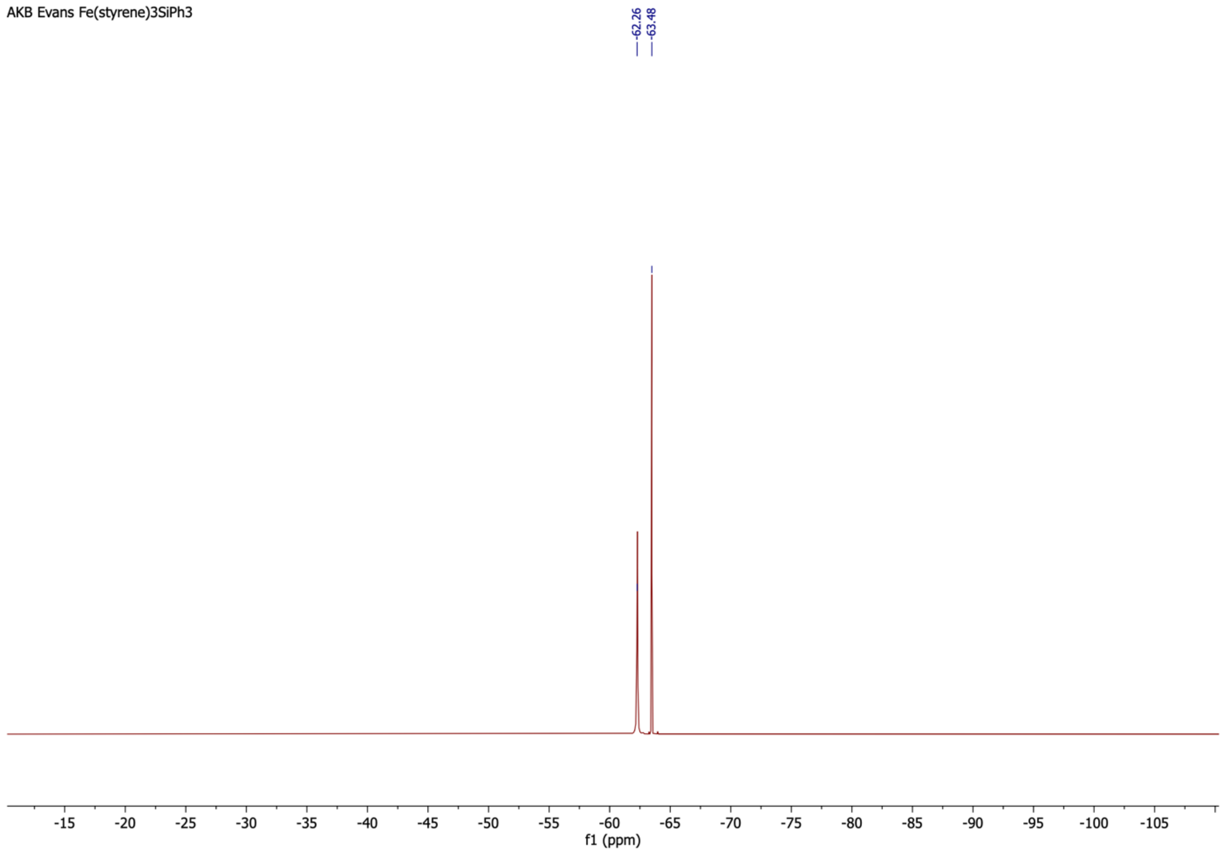

Figure S5. Magnetic Susceptibility Evans Method NMR of crystalline $Fe(styrene)_3SiPh_3$.

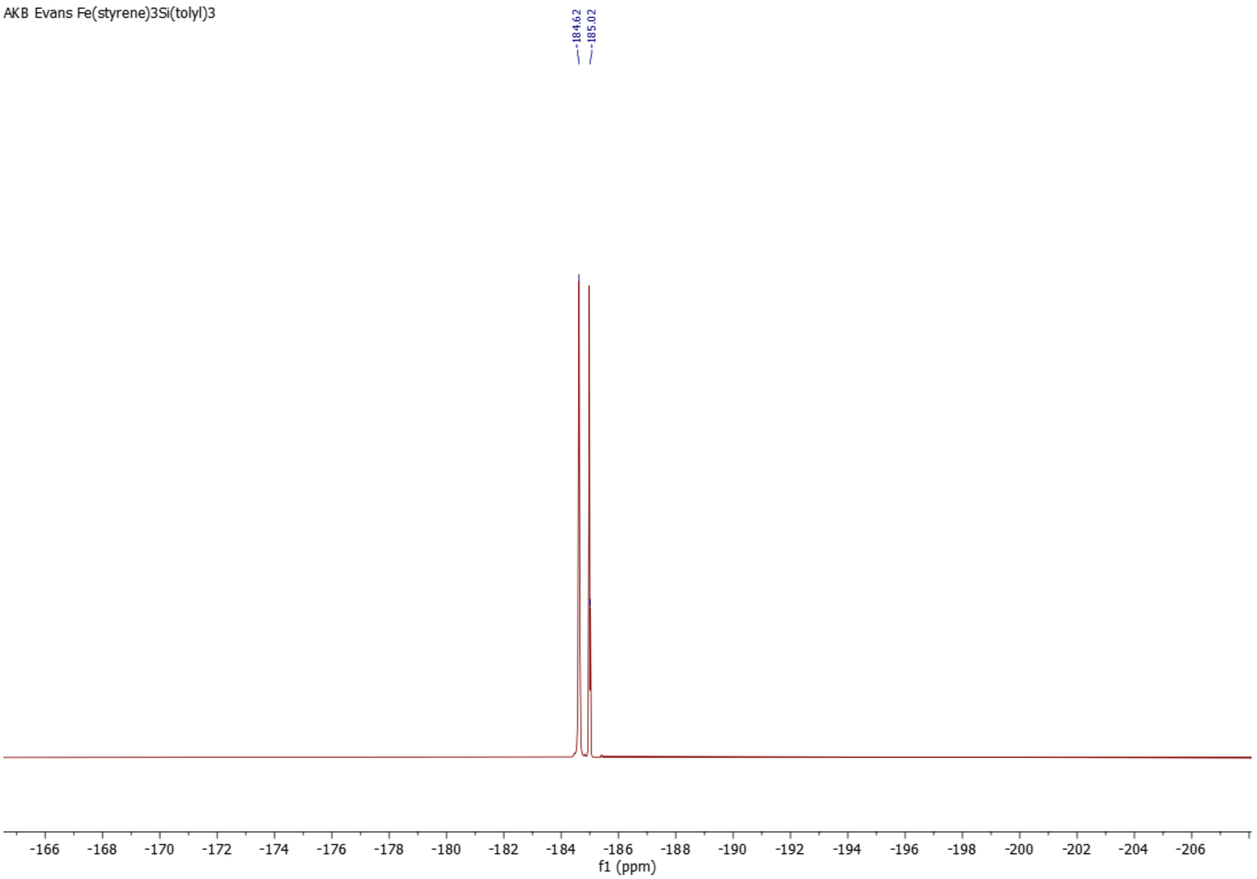

Figure S6. Magnetic Susceptibility Evans Method NMR of crystalline $Fe(styrene)_3Si(tolyl)_3$.

2.2 UV-vis Spectroscopy Data

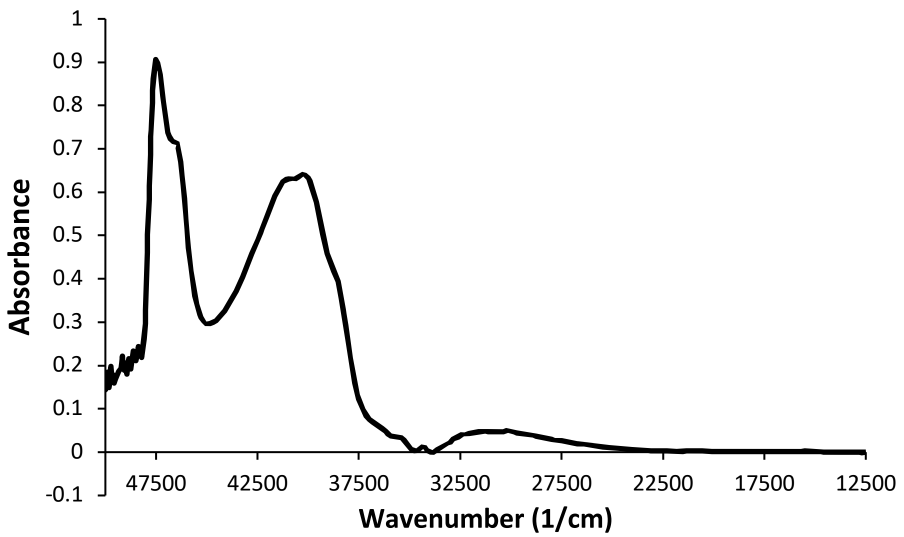

Figure S7. UV-vis spectra of **Fe(styrene)$_3$Et**.

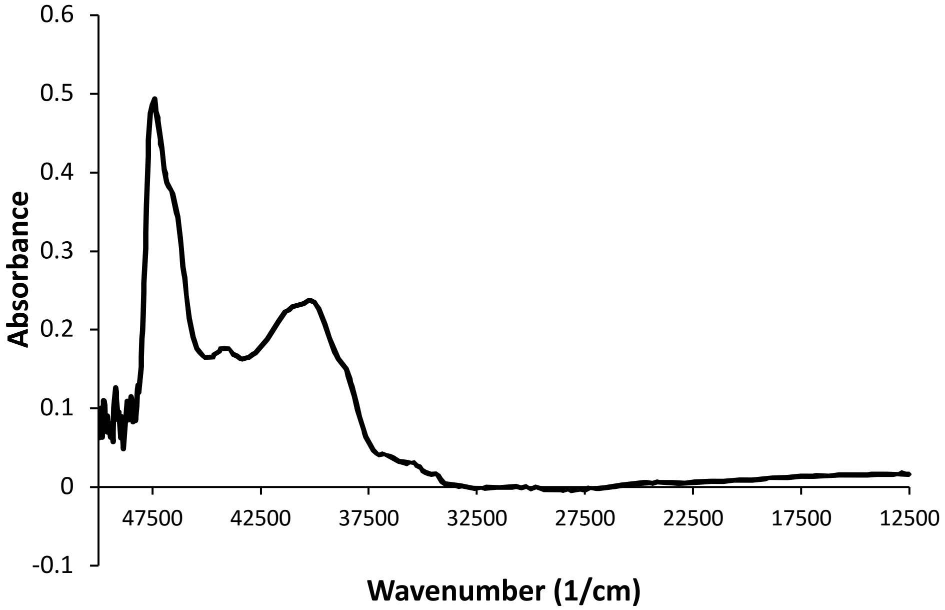

Figure S8. UV-vis spectra of **Fe(styrene)$_3$SiPh$_3$**.

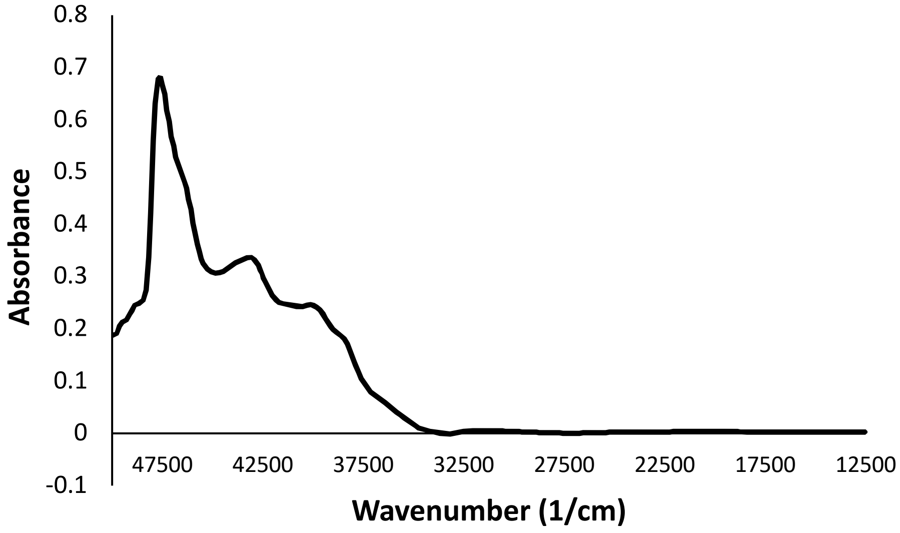

Figure S9. UV-vis spectra of **Fe(styrene)$_3$Si(*p*-tolyl)$_3$**.

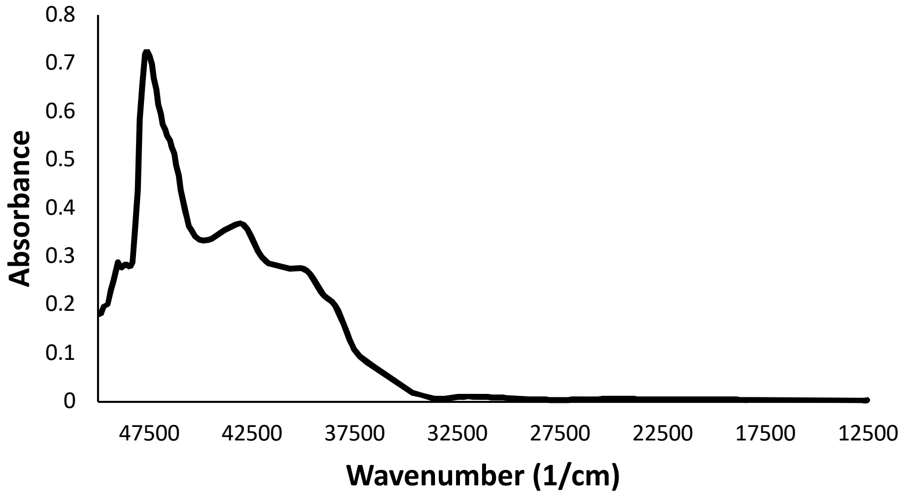

Figure S10. UV-vis spectra of **Fe(styrene)$_3$Si(4-methoxyphenyl)$_3$**.

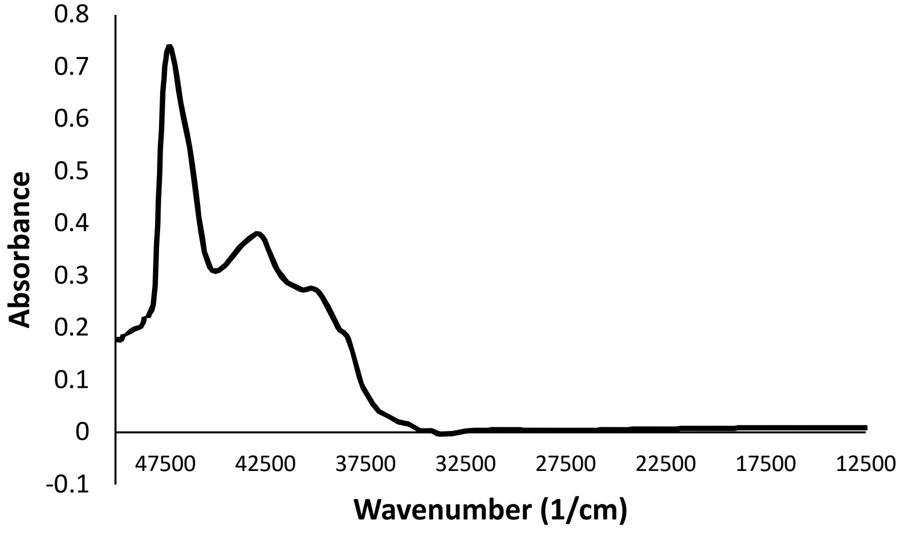

Figure S11. UV-vis spectra of **Fe(styrene)$_3$Si(4-*tert*-butylphenyl)$_3$**.

3. Computational Analysis

3.1 Computational details

All calculations were performed with the ORCA (Revision 6.0.1) program[54]-[56], and all visualisations were generated using ChimeraX 10.01.[55],[56]

Geometry Optimisation and Frequency Calculations

We investigated the triplet electronic state, as this description was found to best reproduce the Mössbauer parameters, accurately reflect the experimental structures, and remain consistent with the Evans NMR measurements. Geometry optimisations were therefore performed at the triplet spin multiplicity using the TPSSh functional,[59],[60] in combination with the x2c-TZVPall basis set.[61] Scalar relativistic effects were included via the exact-two-component (X2C) Hamiltonian,[62]-[64] and the RIJCOSX approximation was employed to accelerate integral evaluation.[54],[65] Dispersion interactions were treated with the D3 dispersion correction together with Becke–Johnson damping.[66],[67] Tight SCF convergence criteria and a dense integration grid (DEFGRID2) were applied throughout. Frequency calculations at the same level of theory confirmed the nature of the stationary points, with all optimised structures displaying no imaginary frequencies. The optimised structures were found to be in close agreement with those obtained from single-crystal X-ray diffraction. All orbital visualisations presented below were obtained from calculations performed at this level of theory.

Mössbauer Parameters

Mössbauer parameters were computed as single-point calculations on the optimised geometries using the wB97X range-separated hybrid functional[68] with the x2c-TZVPPall basis set.[61] The RIJCOSX approximation was employed,[52,64] and scalar relativistic effects were again included via the X2C Hamiltonian.[61]-[63] To improve accuracy, a finite nucleus model was used for iron, together with an auxiliary CP(PPP) basis set.[69] The resulting electron densities were calibrated against the fitting procedure established by Neese et al.,[70] yielding calculated isomer shifts in line with experimental data.

Simulated Spectra

Infrared (IR) and ultraviolet–visible (UV–Vis) absorption spectra were simulated to provide theoretical insight into the electronic and vibrational characteristics of the complexes. All simulations were carried out at the same TPSSh/x2c-TZVPall level of theory used for geometry optimisations.

Electronic absorption spectra were obtained using time-dependent density functional theory (TD-DFT) at the same level of theory. The first 50 excited states (roots) were computed to capture both low-energy d–d and higher-energy charge-transfer transitions. The resulting spectra were generated by convolution of the calculated excitation energies and oscillator strengths using Gaussian broadening (FWHM = 0.18 eV) and uniformly shifted by 20000 cm^{-1} to facilitate comparison of relative transition energies. Analysis of the computed transitions revealed predominantly metal-to-ligand charge-transfer (MLCT) and ligand-centred π–π* excitations within the experimentally relevant energy range.

3.1.1 Fe(Styrene)$_3$Et

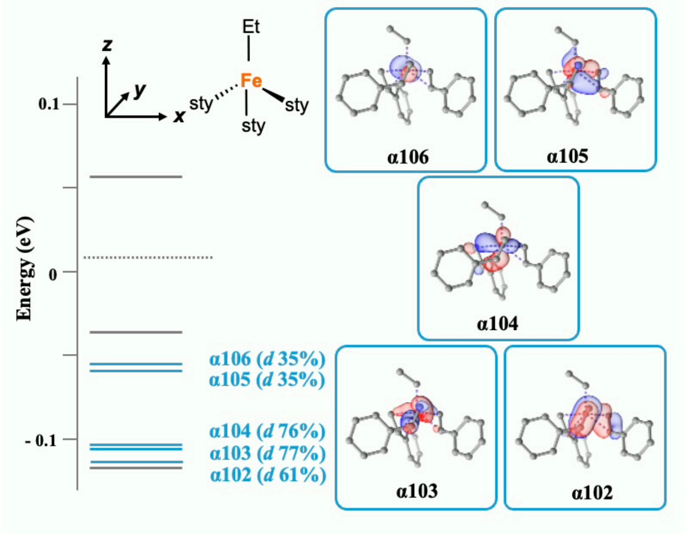

Figure S12. Calculated molecular orbitals (α) for Fe(Styrene)$_3$Et.

Table 1. Percentage (%) of d-orbital character for α molecular orbitals.

**α orbital**	**102**	**103**	**104**	**105**	**106**
dz^2	60.8	0.3	0	0	0
dxz	0.1	56.4	18.4	0.3	1.2
dyz	0.2	18.3	55.1	1.9	0.5
dx^2-y^2	0	0.7	1.7	9.5	23.4
dxy	0.1	0.8	0.7	23.5	9.7
∑	**61.2**	**76.5**	**75.9**	**35.2**	**34.8**

Table 2. Percentage (%) of d-orbital character for β molecular orbitals.

**β orbital**	**103**	**104**	**105**	**106**	**107**
dz^2	0.2	0	20.8	0	0
dxz	24.7	9.1	0.2	9.7	16.9
dyz	9.4	23.1	0	16.3	8.5
dx^2-y^2	1.1	19.6	0	2.5	1.4
dxy	17.3	1.1	0.1	3.1	2
∑	**52.7**	**52.9**	**21.1**	**31.6**	**28.8**

3.1.2 Fe(Styrene)$_3$SiPh$_3$

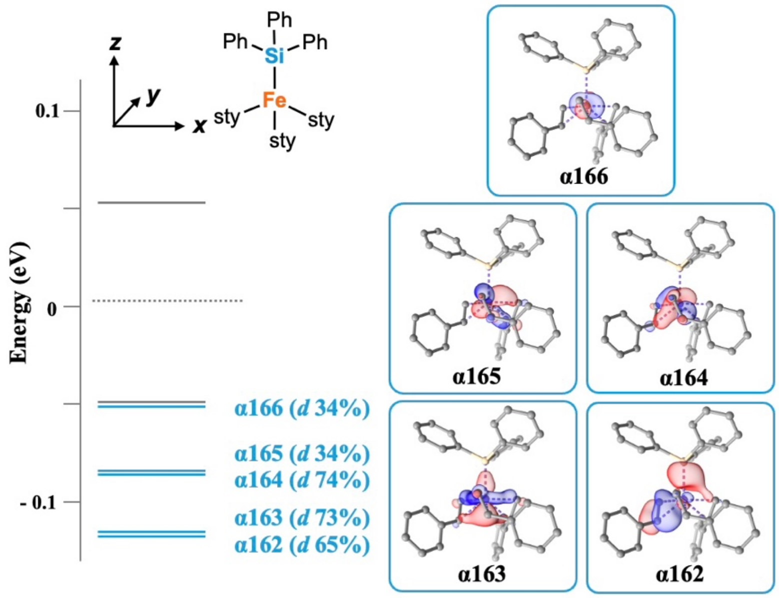

Figure S13. Calculated molecular orbitals (α) for Fe(Styrene)$_3$SiPh$_3$.

Table 3. Percentage (%) of d-orbital character for α molecular orbitals.

**α orbital**	**166**	**165**	**164**	**163**	**162**
dz^2	0	0	0.8	1.1	62.3
dxz	1.2	0.2	62.1	9.6	1.8
dyz	0.1	1.4	10.5	62.3	0.4
dx^2-y^2	18	14.1	0.2	0.2	0
dxy	14.4	18	0	0.2	0
∑	**33.7**	**33.7**	**73.6**	**73.4**	**64.5**

Table 4. Percentage (%) of d-orbital character for β molecular orbitals.

**β orbital**	**163**	**164**	**165**	**166**	**167**
dz^2	0.8	0	29.5	0	0
dxz	12.9	5.1	0	33.7	33.7
dyz	4	16.3	0	5.3	32.5
dx^2-y^2	15	10	0	12.6	1.9
dxy	9.3	15.2	0	1.4	12.6
∑	**42**	**46.6**	**29.5**	**53**	**80.7**

3.1.2 Fe(Styrene)$_3$Si(*p*-tolyl)$_3$

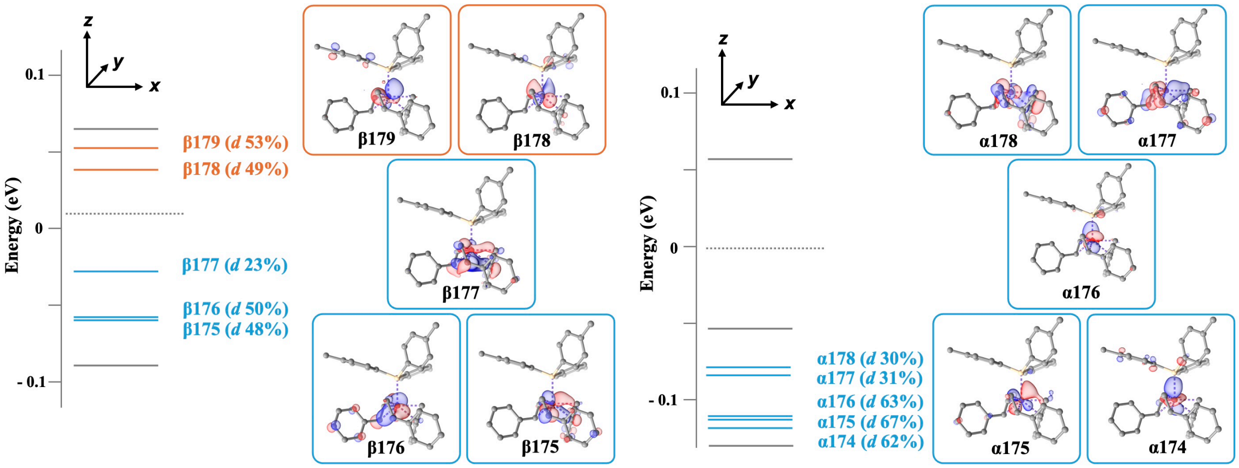

Figure S14. Calculated molecular orbitals (β left, α right) for Fe(Styrene)$_3$Si(*p*-tolyl)$_3$.

Table 5. Percentage (%) of d-orbital character for α molecular orbitals.

**α orbital**	**174**	**175**	**176**	**177**	**178**
dz^2	30.2	5.8	21.4	0	0
dxz	4.3	60.7	1.4	0.5	0.1
dyz	26.9	0.4	38.6	1.2	1.2
dx^2-y^2	0	0.2	0.4	1.9	26.2
dxy	0.1	0.2	1.2	27.3	2.3
∑	**61.5**	**67.3**	**63**	**30.9**	**29.8**

Table 6. Percentage (%) of d-orbital character for β molecular orbitals.

**β orbital**	**175**	**176**	**177**	**178**	**179**
dz^2	0.1	0	23.2	0.4	0.5
dxz	21	13.7	0	22.7	1.3
dyz	12.7	26.5	0	1.6	15.3
dx^2-y^2	8.2	4.7	0.1	12	17.3
dxy	5.9	4.7	0.1	12	18.1
∑	**47.9**	**49.6**	**23.4**	**48.7**	**52.5**

3.1.2 Fe(Styrene)$_3$Si(4-methoxyphenyl)$_3$

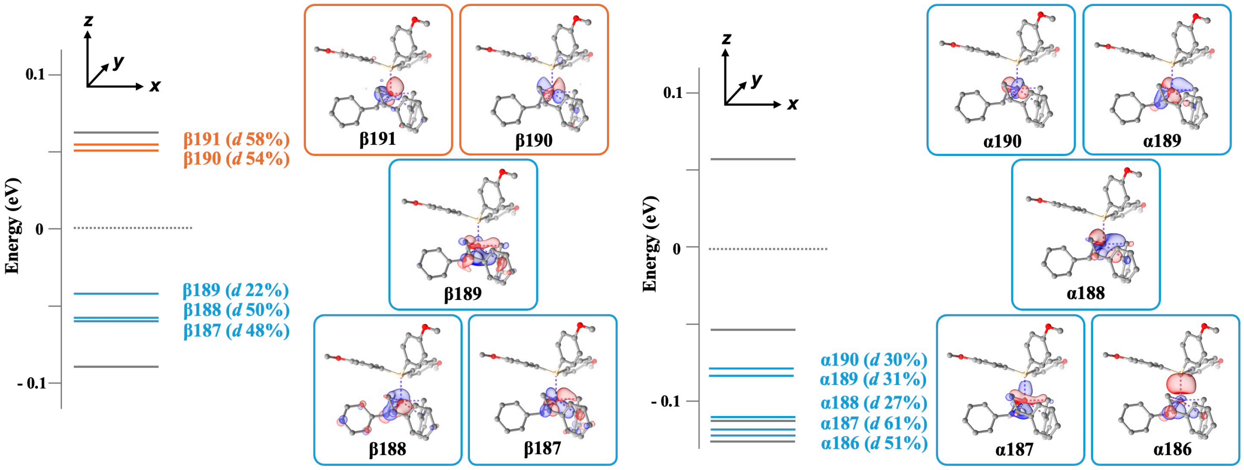

Figure S15. Calculated molecular orbitals (β left, α right) for Fe(Styrene)$_3$Si(4-methoxyphenyl)$_3$.

Table 7. Percentage (%) of d-orbital character for α molecular orbitals.

**α orbital**	**186**	**187**	**188**	**189**	**190**
dz^2	1	7.6	24.8	0	0
dxz	36.2	4.5	1.1	0.9	0.1
dyz	23.4	7.8	1.2	0.5	1.3
dx^2-y^2	0.5	0.3	0	4.3	24
dxy	0.2	0.1	0.1	25.3	4.5
∑	**61.3**	**20.3**	**27.2**	**31**	**29.9**

Table 8. Percentage (%) of d-orbital character for β molecular orbitals.

**β orbital**	**187**	**188**	**189**	**190**	**191**
dz^2	0	0	22	0.1	0.3
dxz	28.6	5.2	0	27.5	0.2
dyz	5.1	34.7	0	0.2	20.4
dx^2-y^2	5.4	6.5	0	6.8	26
dxy	8.8	3.2	0.1	18.9	10.7
∑	**47.9**	**49.6**	**22.1**	**53.5**	**57.6**

3.1.2 Fe(Styrene)$_3$Si(4-tert-butylphenyl)$_3$

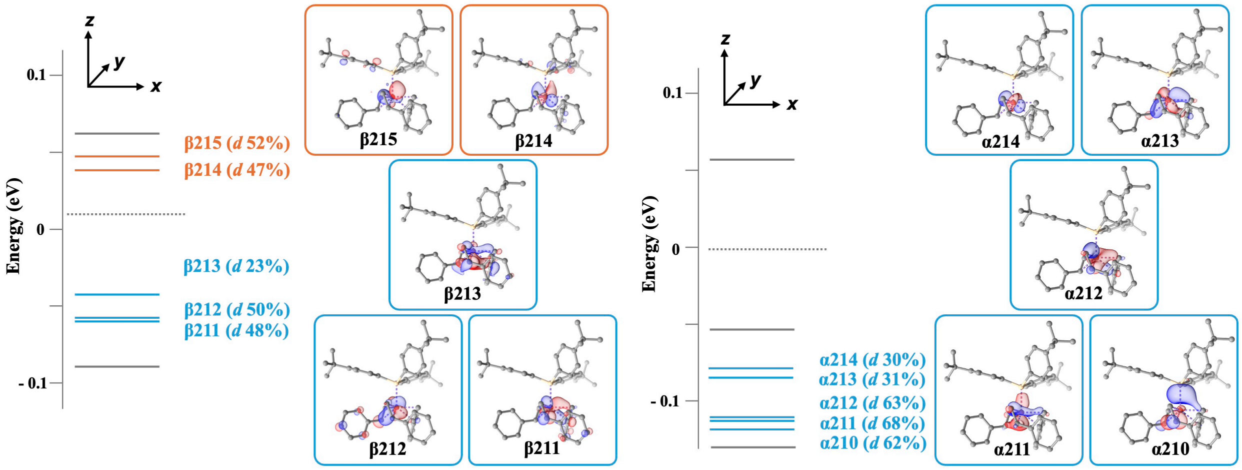

Figure S16. Calculated molecular orbitals (β left, α right) for Fe(Styrene)$_3$Si(4-tert-butylphenyl)$_3$.

Table 9. Percentage (%) of d-orbital character for α molecular orbitals.

α orbital	210	211	212	213	214
dz^2	32.1	2.9	22.8	0	0
dxz	4.5	61.9	0	0.5	0.1
dyz	25	2.6	38.5	1.2	1.2
dx^2-y^2	0	0.2	0.3	3	25.1
dxy	0.1	0.3	1	26.2	3.5
∑	**61.7**	**67.9**	**62.6**	**30.9**	**29.9**

Table 10. Percentage (%) of d-orbital character for β molecular orbitals.

β orbital	211	212	213	214	215
dz^2	0.1	0	23.2	0.4	0.6
dxz	19.6	15.1	0	22.2	1.1
dyz	14.3	24.8	0	1.4	15.5
dx^2-y^2	7.1	5.8	0.1	11.2	18.1
dxy	7	3.9	0.1	12.2	16.6
∑	**48.1**	**49.6**	**23.4**	**47.4**	**51.9**

3.1.3 Fe(Styrene)$_2$SIMes

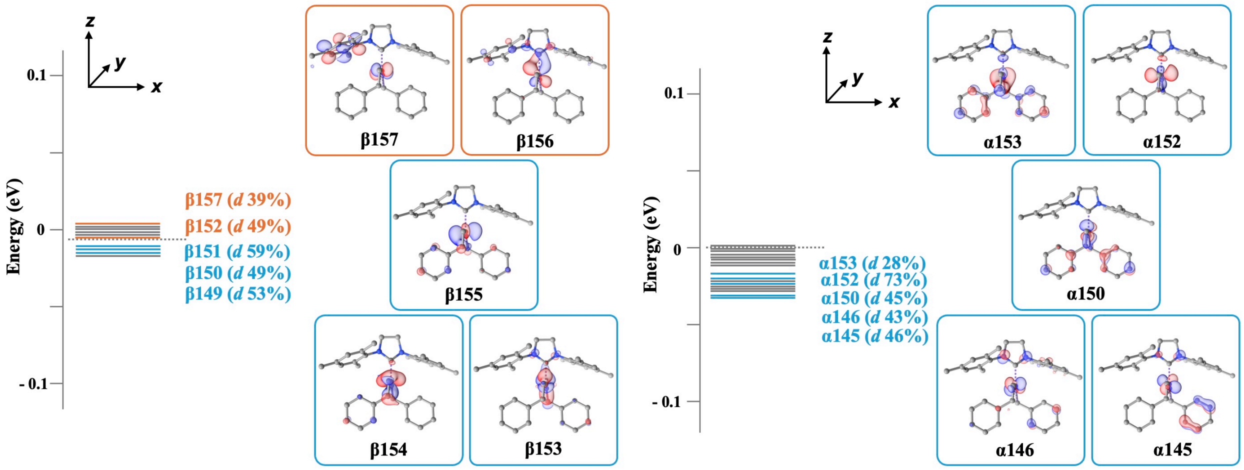

Figure S17. Calculated molecular orbitals (β left, α right) for Fe(Styrene)$_2$SIMes.

Table 11. Percentage (%) of d-orbital character for α molecular orbitals.

**α orbital**	**145**	**146**	**150**	**152**	**153**
dz^2	3.9	0.6	0.2	38.2	1.9
dxz	26	3.5	4.3	2.7	0.5
dyz	2.6	0.3	40.8	0.1	0
dx^2-y^2	0.1	2.7	0	31.1	25.3
dxy	13.3	36.3	0.1	0.5	0
∑	**45.9**	**43.4**	**45.4**	**72.6**	**27.7**

Table 12. Percentage (%) of d-orbital character for β molecular orbitals.

**β orbital**	**153**	**154**	**155**	**156**	**157**
dz^2	2.8	37.3	1.4	0.3	0
dxz	0.3	1.4	3.9	44.1	0.4
dyz	48.6	4.2	0	0.4	0.1
dx^2-y^2	0.9	5.5	53.4	2.7	0
dxy	0.4	0.2	0.6	1.3	38.3
∑	**53**	**48.6**	**59.3**	**48.8**	**38.8**

3.1.4 Mayer Bond Orders

Table 13. Calculated Mayer Bond Orbitals for 1, 2, and Fe(styrene)$_2$SIMes.

**Fe(0)(styrene)$_3$Et (1)**		**Fe(0)(styrene)$_3$SiPh$_3$ (2)**		**Fe(styrene)$_2$SIMes**	
Fe-Et	0.45	Fe-Si	0.66	Fe-NHC	0.50
Fe-styrene (avg)	0.44	Fe-styrene (avg)	0.42	Fe-styrene (avg)	0.46

3.1.5 Calculated IR Stretches

Table 14. Calculated IR stretches for C=C bonds (1/cm).

	**1**	**2**	**3**	**4**	**5**	**Fe(styrene)$_2$SIMes**
**C=C (1)**	1,531.08	1,527.1	1,526.69	1,526.29	1,524.96	1,526.27
**C=C (2)**	1,531.68	1,528.3	1,527.4	1,527.47	1,525.87	1,528.42
**C=C (3)**	1,533.42	1,528.65	1,527.99	1,527.99	1,526.83	

3.1.6 Simulated UV-vis Spectra

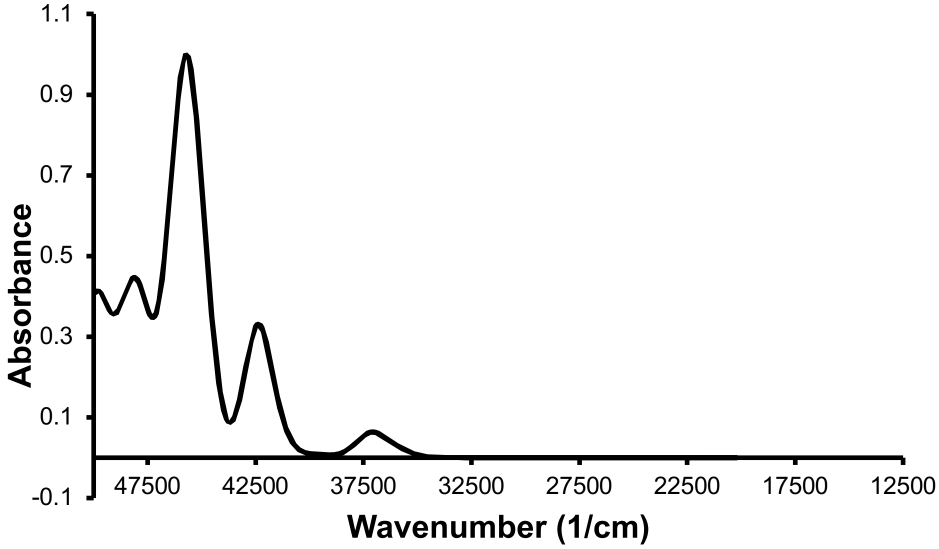

Figure S18. Simulated UV-vis spectrum for **Fe(0)(styrene)$_3$Et.** Calculated peaks (1/cm): 36,925; 42,230; 45,593; 48,032.

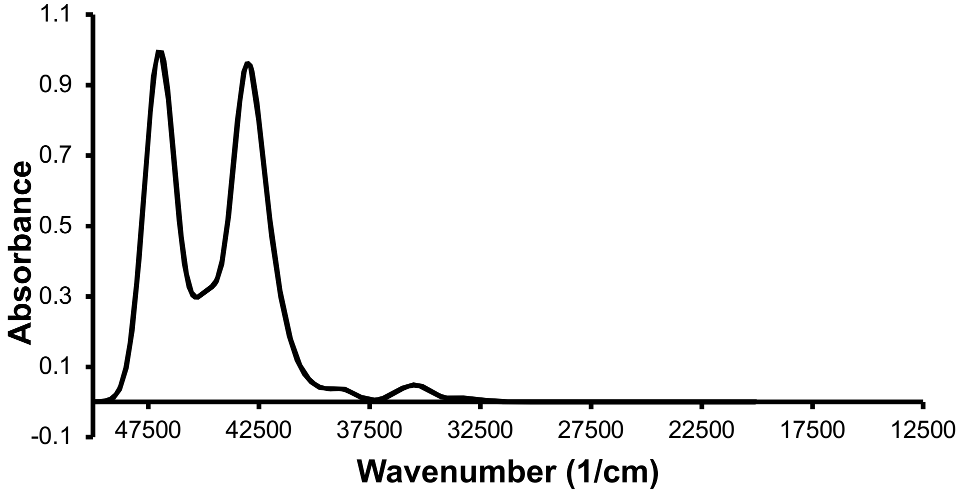

Figure S19. Simulated UV-vis spectrum for **Fe(0)(styrene)$_3$SiPh$_3$.** Calculated peaks (1/cm): 42,854; 46,981.

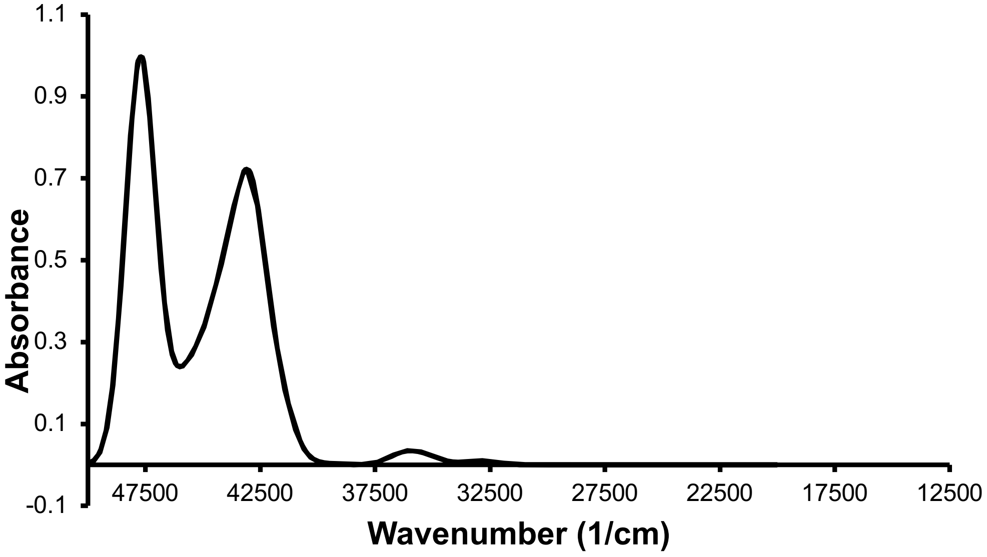

Figure S20. Simulated UV-vis spectrum for **Fe(0)(styrene)$_3$Si(*p*-tolyl)$_3$.** Calculated peaks (1/cm): 42,914; 47,578.

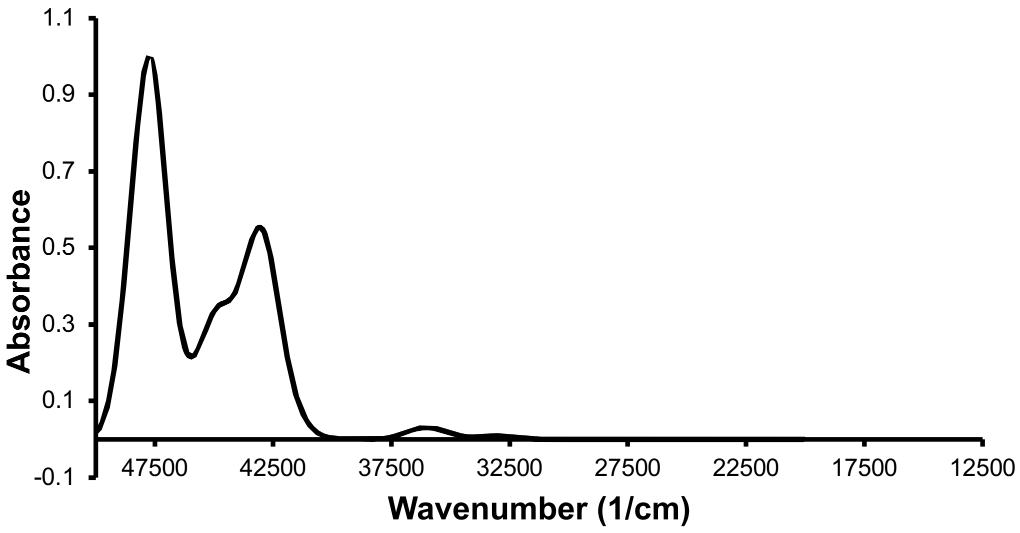

Figure S21. Simulated UV-vis spectrum for **Fe(0)(styrene)$_3$Si(4-methoxyphenyl)$_3$.** Calculated peaks (1/cm): 42,935; 44,776; 47,676.

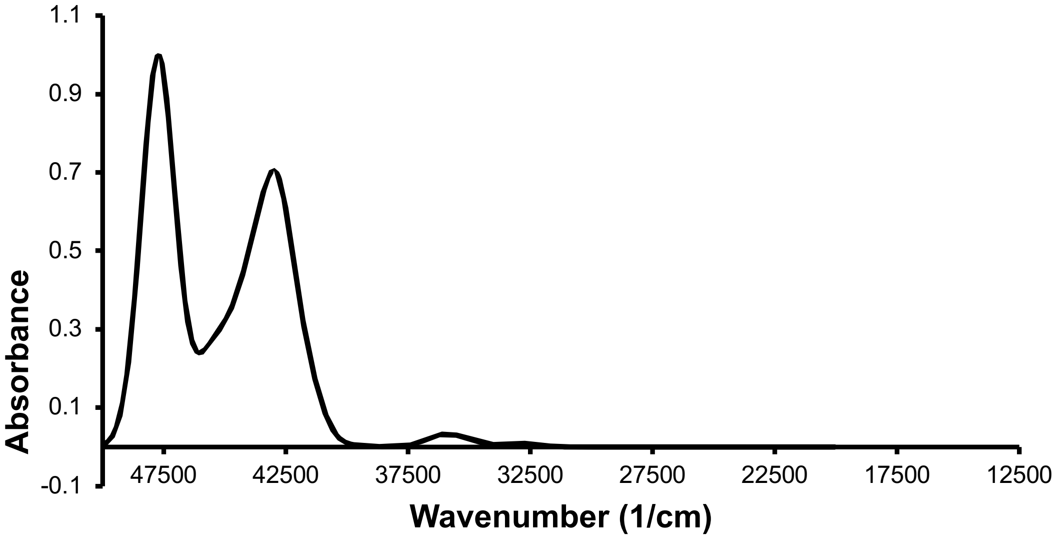

Figure S22. Simulated UV-vis spectrum for **Fe(0)(styrene)$_3$Si(4-tert-butylphenyl)$_3$.** Calculated peaks (1/cm): 42,849; 47,630

4. X-ray Crystallographic Data

Single-crystal X-ray diffraction data was collected on an Oxford Diffraction/Agilent SuperNova diffractometer equipped with a 135 mm Atlas CCD area detector. The crystal was selected under a nitrogen atmosphere, mounted on a MicroMesh sample pin and cooled using an Oxford Cryosystems open flow N_2 cooling device. Data was collected at 150 K using mirror monochromated Cu K(α) radiation (wavelength = 1.5418 Å; Oxford Diffraction Supernova). Data was then processed using CrysAlisPro package[77], structures solved ab initio from the integrated intensities using SHELXT[78] and refined using SHELXL[79] with the graphical interface OLEX2.[80]

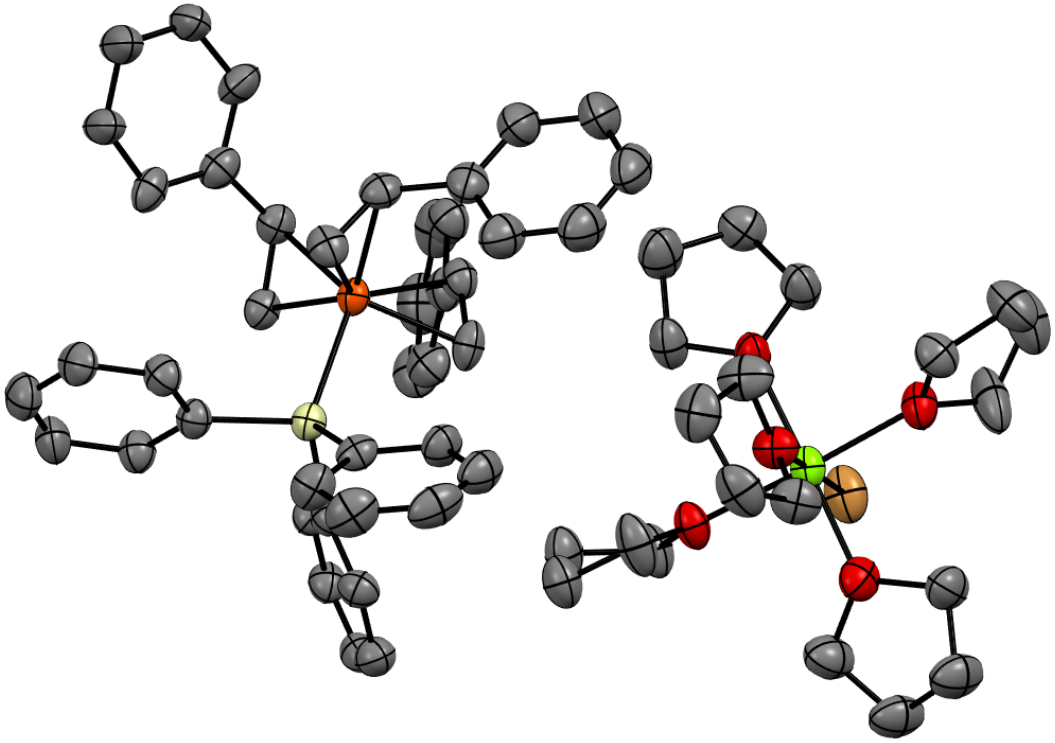

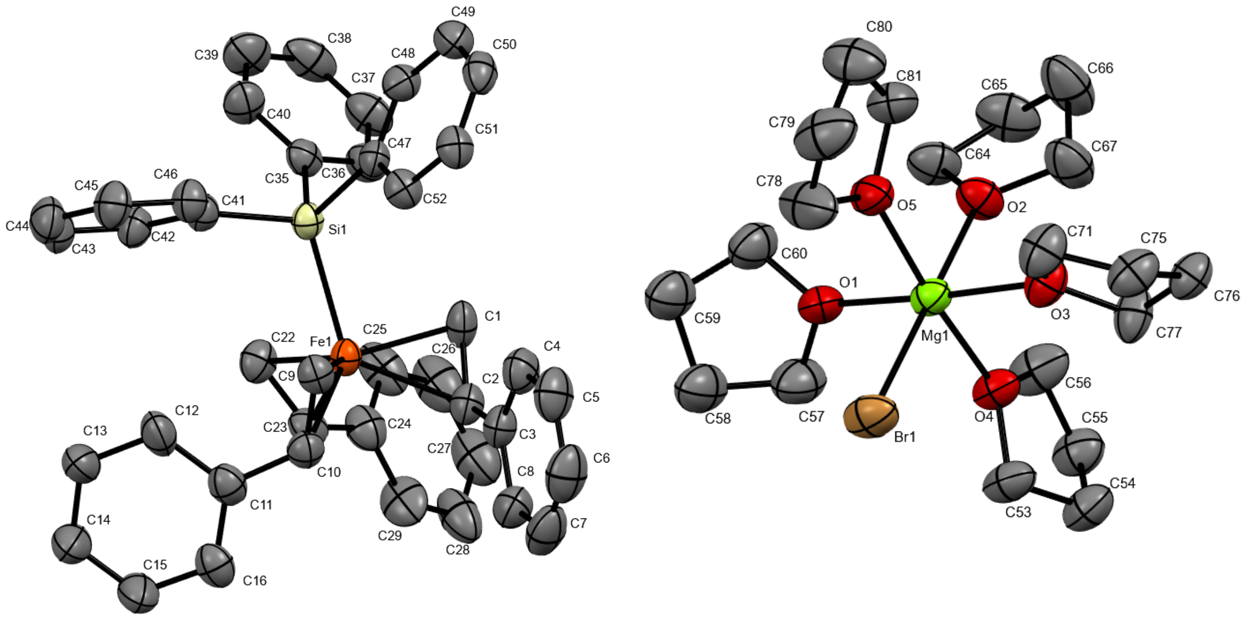
C80
C81
C49
C50
C38
C39
C48
C37
C51
C40
C47
C35
C36
C52
C46
C45
C41
C44
C43
C42
Si1
C65
C66
C79
C64
C67
C78
O5
O2
C71
C75
C60
O1
C76
O3
C77
Mg1
C59
C1
C25
C22
Fe1
C26
C4
C9
C2
C5
C23
C24
C3
C6
C27
C8
C10
C11
C7
C12
C13
C14
C15
C16
C29
C28
C56
C55
C57
C58
O4
Br1
C54
C53

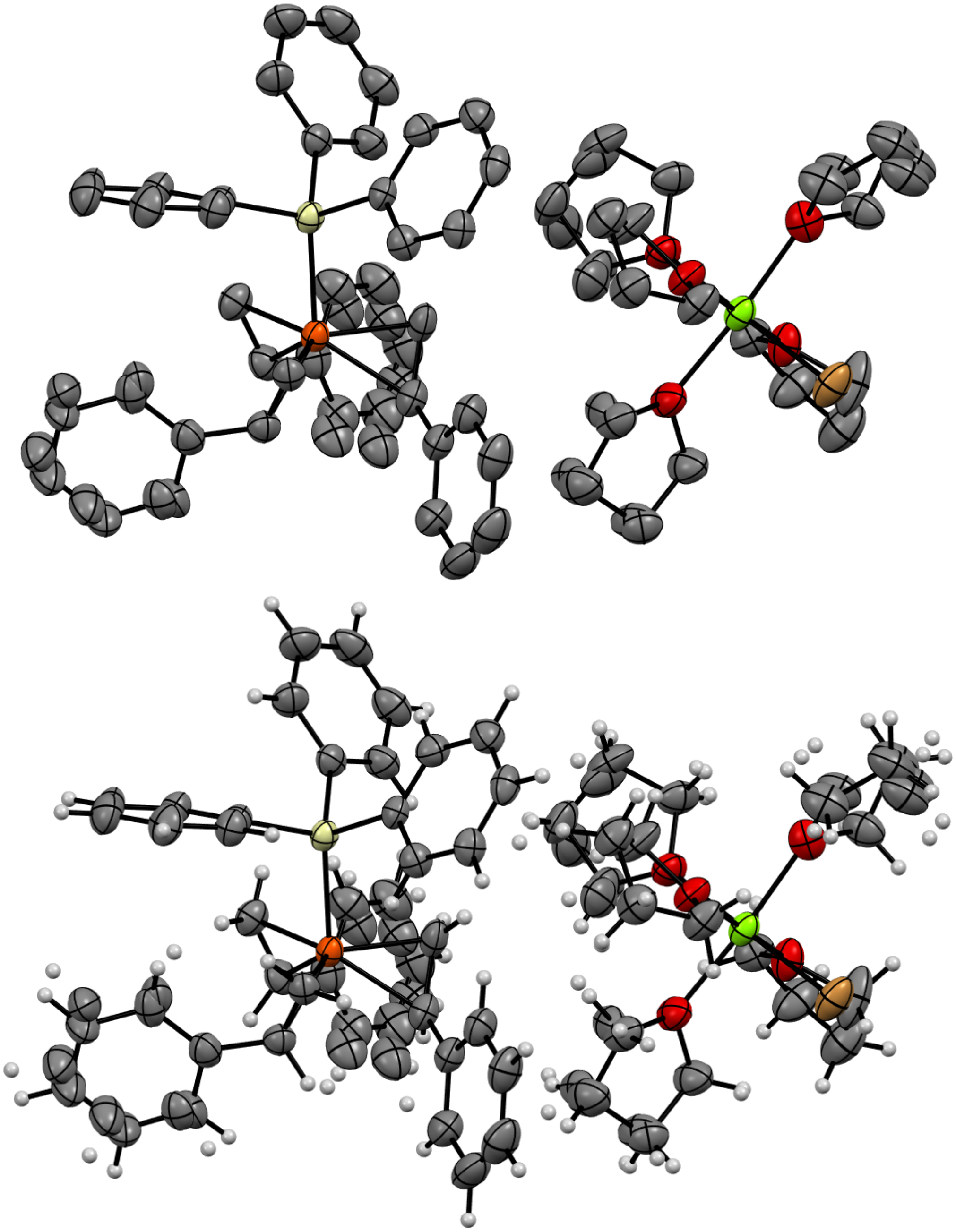

Reference number: 027akb24

CCDC number: 2484414

Crystal Structure Report: **Fe(styrene)$_3$SiPh$_3$**

Table 15. Crystal Data and structure refinement for **Fe(styrene)$_3$SiPh$_3$**.

Identification code	027akb24	
Empirical formula	C68 H92 Br Fe Mg O5 Si	
Formula weight	1178.38	
Temperature	150.01(10) K	
Wavelength	Cu Kα (λ = 1.54184)	
Crystal system	triclinic	
Space group	P-1	
Unit cell dimensions	*a* = 12.9700(6) Å	α = 83.348(4)°
	b = 13.7887(5) Å	β = 70.267(4)°
	c = 18.9046(9) Å	γ = 88.763(4)°
Volume	3160.4(3) Å^3	
Z	2	
Density (calculated)	1.238 mg/m^3	
Absorption coefficient	3.295 mm^{-1}	
F(000)	1256	
Crystal color, morphology	orange, block	
Crystal size	0.4 x 0.1 x 0.1 mm^3	
Theta range for data collection	7.24 to 156.18°	
Index ranges	$-16 \le h \le 16$, $-14 \le k \le 17$, $-23 \le l \le 23$	
Reflections collected	69259	
Independent reflections	13112[*R*(int) = 0.1092]	
Observed reflections	38462	
Completeness to theta = 25.242°	100.0%	
Absorption correction	Multi-scan	
Max. and min. transmission	1.00 and 0.090	
Refinement method	Full-matrix least-squares on F^2	
Data / restraints / parameters	13112 / 384 / 724	
Goodness-of-fit on *F*2	0.998	
Final *R* indices [*I*>2sigma(*I*)]	*R*1 = 0.0552, *wR*2 = 0.1357	
R indices (all data)	*R*1 = 0.0927, *wR*2 = 0.1562	
Largest diff. peak and hole	0.42 and -0.79 e.Å^{-3}	

Table 16. Atomic coordinates (x 10^4) and equivalent isotropic displacement parameters ($Å^2$ x 10^3) for 027akb24. U_{eq} is defined as one third of the trace of the orthogonalized U_{ij} tensor.

Atom	*x*	*y*	*z*	U(eq)
Br1	-100.5(3)	2768.1(4)	3832.4(3)	61.38(15)
Fe1	5290.1(4)	8077.5(4)	2233.9(3)	33.53(13)
Si1	6019.4(7)	7810.5(6)	3239.7(5)	34.21(19)
Mg1	1992.1(9)	2833.5(8)	3269.1(7)	39.8(3)
O4	2123(2)	3846.9(18)	4002.3(16)	45.6(6)
O2	3743(2)	2882.7(19)	2816.1(15)	48.2(6)
O3	2007(2)	4008.3(19)	2452.4(17)	50.4(6)
O1	2122(2)	1676.0(19)	4053.2(16)	51.0(6)
O5	2030(2)	1844.8(19)	2475.4(16)	51.1(6)
C9	4462(3)	9257(2)	2728(2)	39.1(7)
C47	4978(3)	7396(2)	4211.3(19)	35.8(7)
C22	6917(3)	8231(3)	1537(2)	45.3(8)
C48	5154(3)	6638(3)	4704(2)	44.0(8)
C35	7116(3)	6850(3)	3159(2)	40.0(7)
C10	4627(3)	9472(2)	1947(2)	41.3(8)
C1	4532(3)	6707(2)	2527(2)	46.5(9)
C42	7713(3)	9308(3)	2849(2)	43.8(8)
C41	6686(3)	8981(2)	3352(2)	39.0(7)
C51	3137(3)	7507(3)	5141(2)	43.5(8)
C46	6121(3)	9607(3)	3879(2)	44.4(8)
C50	3348(3)	6757(3)	5609(2)	47.0(9)
C49	4358(3)	6313(3)	5397(2)	48.9(9)
C52	3936(3)	7820(3)	4458(2)	41.7(8)
C11	5425(3)	10199(3)	1444(2)	47.9(8)

Atom	*x*	*y*	*z*	U(eq)
C23	6339(3)	8026(3)	1062(2)	43.9(8)
C2	3948(3)	7266(3)	2113(2)	43.8(8)
C36	6988(3)	5944(3)	2924(2)	49.8(9)
C40	8054(3)	6965(3)	3354(3)	52.5(10)
C38	8635(4)	5333(3)	3105(3)	63.1(12)
C45	6563(4)	10521(3)	3892(3)	53.8(10)
C43	8150(3)	10225(3)	2865(2)	51.1(9)
C53	1225(3)	4378(3)	4490(3)	56.5(10)
C3	2845(3)	7650(3)	2459(2)	44.9(8)
C39	8804(4)	6214(3)	3324(3)	62.8(12)
C4	2309(3)	7600(3)	3239(3)	53.6(10)
C12	6280(20)	10530(20)	1685(13)	58(6)
C81	2959(3)	1671(3)	1818(2)	53.8(9)
C75	1430(40)	4950(11)	1540(30)	55(5)
C64	4466(3)	2219(3)	3067(3)	55.9(10)
C16	5400(20)	10610(20)	739(10)	49(4)
C5	1258(3)	7954(3)	3541(3)	63.4(12)
C37	7738(4)	5199(3)	2900(3)	58.0(11)
C7	1216(4)	8423(3)	2302(3)	66.8(13)
C8	2269(3)	8083(3)	1997(3)	55.0(10)
C44	6770(40)	6240(20)	890(30)	61(6)
C54	7576(4)	10827(3)	3387(3)	56.1(10)
C55	1754(4)	5269(3)	4615(3)	59.4(11)
C6	2853(4)	4890(3)	4634(3)	59.6(11)
C71	715(4)	8358(4)	3076(3)	70.0(13)
C15	1533(4)	3900(3)	1860(3)	59.1(11)

Atom	*x*	*y*	*z*	U(eq)
C56	5690(40)	7120(30)	240(20)	59(6)
C80	6190(20)	11306(18)	307(13)	64(5)
C57	3140(4)	4149(4)	4088(3)	69.2(14)
C65	2496(4)	1153(4)	1322(3)	71.9(13)
C58	1771(5)	1731(4)	4855(3)	68.1(12)
C77	5557(10)	2765(16)	2709(11)	71(3)
C78	1110(20)	765(13)	5138(15)	70(3)
C76	2280(30)	5035(11)	2454(16)	55(5)
C13	1096(4)	1273(4)	2453(3)	86.9(18)
C60	2280(30)	5509(12)	1702(14)	49(4)
C67	6987(16)	11306(17)	1285(17)	54(4)
C59	2610(20)	706(12)	4045(14)	63(3)
C79	4348(8)	3668(10)	2229(8)	65(3)
C14	5560(30)	6320(30)	-81(18)	64(6)
C66	1720(20)	66(14)	4594(16)	67(3)
C63	1295(4)	1189(4)	1662(4)	81.6(16)
C62	6690(40)	5450(20)	540(20)	66(6)
C61	6160(30)	5490(30)	20(20)	69(7)
C68	6970(17)	11665(16)	572(16)	62(5)
C69	5504(8)	3292(11)	1981(9)	83(4)
C70	2019(6)	655(4)	3939(3)	58.4(15)
C74	2124(7)	98(4)	4639(4)	69.8(17)
C73	1515(7)	730(4)	5245(4)	68.3(16)
C72	5585(16)	2640(20)	2882(19)	65(5)
C30	5559(11)	3506(12)	2335(14)	68(4)
C31	4552(14)	3373(18)	2134(10)	65(5)

Atom	*x*	*y*	*z*	U(eq)
C32	1933(18)	5042(8)	2566(11)	56(3)
C33	1950(20)	5563(9)	1826(13)	70(4)
C34	1270(20)	4914(9)	1564(17)	57(3)
C24	5898(10)	6869(7)	272(6)	60.8(19)
C29	5930(9)	5984(8)	-2(5)	71(3)
C28	6501(9)	5228(6)	250(6)	71(3)
C27	7010(8)	5387(5)	754(6)	69(2)
C26	6969(10)	6268(6)	1026(7)	58.9(18)
C25	6372(3)	7063(3)	784(2)	52.4(9)
C18	6905(14)	11366(13)	1067(11)	57(3)
C20	6023(13)	11096(13)	172(8)	56(3)
C19	6803(15)	11588(10)	359(11)	69(4)
C17	6199(13)	10709(14)	1635(9)	45(3)
C21	5373(17)	10391(18)	704(8)	56(4)

Table 17. Bond Lengths for 027akb24.

Atom	Atom	Length/Å	Atom	Atom	Length/Å
Br1	Mg1	2.5584(12)	C38	C37	1.366(7)
Fe1	Si1	2.3840(11)	C45	C44	1.378(6)
Fe1	C9	2.067(3)	C43	C44	1.373(6)
Fe1	C22	2.076(3)	C53	C54	1.500(6)
Fe1	C10	2.175(3)	C3	C4	1.395(6)
Fe1	C1	2.078(3)	C3	C8	1.407(6)
Fe1	C23	2.182(4)	C4	C5	1.389(6)
Fe1	C2	2.179(3)	C12	C13	1.395(13)
Si1	C47	1.908(3)	C81	C80	1.518(6)
Si1	C35	1.903(3)	C75	C71	1.525(14)
Si1	C41	1.914(3)	C75	C76	1.499(15)
Mg1	O4	2.126(3)	C64	C65	1.520(10)
Mg1	O2	2.139(3)	C64	C68	1.488(14)
Mg1	O3	2.100(3)	C16	C15	1.391(14)
Mg1	O1	2.093(3)	C5	C6	1.367(7)
Mg1	O5	2.129(3)	C7	C8	1.386(6)
O4	C53	1.462(4)	C7	C6	1.379(8)
O4	C56	1.457(4)	C54	C55	1.354(18)
O2	C64	1.451(4)	C55	C56	1.25(3)
O2	C67	1.480(9)	C71	C72	1.517(6)
O2	C70	1.461(13)	C15	C14	1.489(6)
O3	C71	1.469(5)	C80	C79	1.519(10)
O3	C77	1.467(12)	C57	C58	1.352(18)
O3	C74	1.462(9)	C57	C61	1.56(3)
O1	C57	1.439(5)	C65	C66	1.389(14)

Atom	Atom	Length/Å	Atom	Atom	Length/Å
O1	C60	1.469(15)	C58	C59	1.474(7)
O1	C63	1.464(6)	C77	C76	1.541(17)
O5	C81	1.449(5)	C78	C79	1.476(7)
O5	C78	1.475(5)	C13	C14	1.503(13)
C9	C10	1.414(5)	C60	C59	1.504(17)
C47	C48	1.385(5)	C67	C66	1.492(13)
C47	C52	1.412(5)	C63	C62	1.447(8)
C22	C23	1.403(5)	C62	C61	1.388(14)
C48	C49	1.398(5)	C68	C69	1.490(18)
C35	C36	1.404(5)	C69	C70	1.511(12)
C35	C40	1.402(5)	C74	C73	1.40(2)
C10	C11	1.463(5)	C73	C72	1.37(2)
C1	C2	1.420(5)	C30	C31	1.498(8)
C42	C41	1.398(5)	C30	C24	1.512(8)
C42	C43	1.403(5)	C31	C32	1.492(16)
C41	C46	1.397(5)	C32	C33	1.499(16)
C51	C50	1.367(5)	C33	C34	1.490(11)
C51	C52	1.383(5)	C34	C24	1.499(11)
C46	C45	1.402(5)	C24	C29	1.375(8)
C50	C49	1.386(5)	C24	C25	1.362(9)
C11	C12	1.433(13)	C29	C28	1.398(11)
C11	C16	1.400(12)	C28	C27	1.368(11)
C11	C17	1.404(9)	C27	C26	1.365(8)
C11	C21	1.418(10)	C26	C25	1.447(8)
C23	C24	1.479(5)	C18	C19	1.388(11)
C2	C3	1.474(5)	C18	C17	1.400(11)

Atom	Atom	Length/Å	Atom	Atom	Length/Å
C36	C37	1.394(5)	C20	C19	1.391(11)
C40	C39	1.398(5)	C20	C21	1.380(10)
C38	C39	1.373(7)			

Table 18. Bond Angles for 027akb24.

Atom	Atom	Atom	Angle/°	Atom	Atom	Atom	Angle/°
C9	Fe1	Si1	88.48(10)	C16	C11	C10	124.0(10)
C9	Fe1	C22	120.30(15)	C16	C11	C12	117.1(11)
C9	Fe1	C10	38.85(14)	C17	C11	C10	126.0(7)
C9	Fe1	C1	119.32(14)	C17	C11	C21	119.1(8)
C9	Fe1	C23	128.21(14)	C21	C11	C10	114.9(7)
C9	Fe1	C2	100.63(14)	C22	C23	Fe1	66.7(2)
C22	Fe1	Si1	84.94(11)	C22	C23	C24	122.4(4)
C22	Fe1	C10	101.75(15)	C24	C23	Fe1	114.7(2)
C22	Fe1	C1	120.08(15)	C1	C2	Fe1	66.72(19)
C22	Fe1	C23	38.39(14)	C1	C2	C3	123.9(4)
C22	Fe1	C2	129.72(15)	C3	C2	Fe1	115.6(3)
C10	Fe1	Si1	122.48(10)	C37	C36	C35	121.9(4)
C10	Fe1	C23	91.73(14)	C39	C40	C35	121.7(4)
C10	Fe1	C2	92.16(14)	C37	C38	C39	119.6(4)
C1	Fe1	Si1	91.21(12)	C44	C45	C46	120.9(4)
C1	Fe1	C10	128.83(14)	C44	C43	C42	120.4(4)
C1	Fe1	C23	102.95(15)	O4	C53	C54	105.0(3)
C1	Fe1	C2	38.88(14)	C4	C3	C2	123.6(4)
C23	Fe1	Si1	120.84(10)	C4	C3	C8	116.5(4)
C2	Fe1	Si1	126.71(11)	C8	C3	C2	119.9(4)
C2	Fe1	C23	93.94(15)	C38	C39	C40	120.4(4)
C47	Si1	Fe1	115.26(11)	C5	C4	C3	121.7(4)
C47	Si1	C41	105.73(15)	C13	C12	C11	122.4(14)
C35	Si1	Fe1	117.37(12)	O5	C81	C80	105.8(3)
C35	Si1	C47	101.78(15)	C76	C75	C71	104.3(13)

Atom	Atom	Atom	Angle/°	Atom	Atom	Atom	Angle/°
C35	Si1	C41	104.63(15)	O2	C64	C65	101.2(7)
C41	Si1	Fe1	110.83(11)	O2	C64	C68	112.7(10)
O4	Mg1	Br1	92.59(8)	C15	C16	C11	119.4(13)
O4	Mg1	O2	86.86(11)	C6	C5	C4	120.4(5)
O4	Mg1	O5	174.30(12)	C38	C37	C36	120.4(4)
O2	Mg1	Br1	179.08(10)	C6	C7	C8	120.1(5)
O3	Mg1	Br1	93.19(9)	C7	C8	C3	121.5(5)
O3	Mg1	O4	89.19(11)	C43	C44	C45	126(3)
O3	Mg1	O2	87.54(11)	C53	C54	C55	119.1(4)
O3	Mg1	O5	89.52(12)	C56	C55	C54	102.4(3)
O1	Mg1	Br1	91.63(9)	C5	C6	C7	104.3(3)
O1	Mg1	O4	90.00(12)	O3	C71	C75	119.8(4)
O1	Mg1	O2	87.63(11)	O3	C71	C72	103.7(11)
O1	Mg1	O3	175.15(12)	C14	C15	C16	107.7(8)
O1	Mg1	O5	90.83(12)	O4	C56	C55	121(2)
O5	Mg1	Br1	93.03(8)	C79	C80	C81	122.5(13)
O5	Mg1	O2	87.54(11)	O1	C57	C58	107.0(3)
C53	O4	Mg1	126.4(2)	O1	C57	C61	106.2(4)
C56	O4	Mg1	125.3(2)	C66	C65	C64	99.3(11)
C56	O4	C53	108.3(3)	C59	C58	C57	108.0(4)
C64	O2	Mg1	125.5(2)	O3	C77	C76	104.8(8)
C64	O2	C67	112.6(5)	C79	C78	O5	104.7(14)
C64	O2	C70	98.7(9)	C77	C76	C75	104.0(12)
C67	O2	Mg1	121.7(4)	C14	C13	C12	105.9(4)
C70	O2	Mg1	134.7(8)	O1	C60	C59	104.1(14)
C71	O3	Mg1	120.9(2)	O2	C67	C66	118.2(14)

Atom	Atom	Atom	Angle/°	Atom	Atom	Atom	Angle/°
C77	O3	Mg1	127.4(9)	C60	C59	C58	103.7(14)
C77	O3	C71	110.8(10)	C78	C79	C80	102.5(8)
C74	O3	Mg1	126.1(7)	C13	C14	C15	117(2)
C74	O3	C71	106.5(7)	C65	C66	C67	101.7(15)
C57	O1	Mg1	123.3(2)	O1	C63	C62	104.6(4)
C57	O1	C60	98.4(11)	C63	C62	C61	121(2)
C57	O1	C63	107.8(4)	C57	C61	C62	120(2)
C60	O1	Mg1	138.0(10)	C64	C68	C69	119.6(12)
C63	O1	Mg1	121.9(3)	C68	C69	C70	101.5(11)
C81	O5	Mg1	126.2(2)	O2	C70	C69	103.4(5)
C81	O5	C78	107.0(3)	O3	C74	C73	102.4(4)
C78	O5	Mg1	126.6(3)	C74	C73	C72	103.3(5)
C10	C9	Fe1	74.7(2)	C73	C72	C71	101.0(11)
C48	C47	Si1	123.6(3)	C31	C30	C24	105.9(12)
C48	C47	C52	115.3(3)	C30	C31	C32	105.8(12)
C52	C47	Si1	121.1(3)	C33	C32	C31	104.7(9)
C23	C22	Fe1	74.9(2)	C34	C33	C32	103.7(9)
C47	C48	C49	122.7(4)	C33	C34	C24	102.7(9)
C36	C35	Si1	120.0(3)	C30	C24	C23	124.7(7)
C40	C35	Si1	124.0(3)	C30	C24	C34	118.1(7)
C40	C35	C36	116.0(3)	C34	C24	C23	119.6(6)
C9	C10	Fe1	66.43(18)	C29	C24	C23	121.7(7)
C9	C10	C11	123.6(3)	C29	C24	C25	120.1(7)
C11	C10	Fe1	115.9(2)	C25	C24	C23	108.3(13)
C2	C1	Fe1	74.4(2)	C24	C29	C28	137.2(17)
C41	C42	C43	121.7(4)	C27	C28	C29	114.4(15)

Atom	Atom	Atom	Angle/°	Atom	Atom	Atom	Angle/°
C42	C41	Si1	121.1(3)	C28	C27	C26	123.6(5)
C46	C41	Si1	121.7(3)	C25	C26	C27	115.7(5)
C46	C41	C42	116.7(3)	C26	C25	C24	120.6(5)
C50	C51	C52	119.7(3)	C19	C18	C17	122.8(10)
C41	C46	C45	121.2(4)	C21	C20	C19	118.2(9)
C51	C50	C49	119.9(4)	C18	C19	C20	119.9(9)
C50	C49	C48	119.5(4)	C18	C17	C11	117.2(10)
C51	C52	C47	122.9(3)	C20	C21	C11	122.4(10)
C12	C11	C10	118.8(9)				

Table 19. Anisotropic displacement parameters (Å2x 103) for 027akb24. The anisotropic displacement factor exponent takes the form: -2p2[h2 a*2U11 + ... + 2 h k a* b* U12]

Atom	U*11*	U*22*	U*33*	U*23*	U*13*	U*12*
Br1	34.4(2)	69.2(3)	81.4(3)	-28.7(2)	-14.4(2)	2.48(18)
Fe1	28.3(2)	31.7(2)	39.1(3)	-5.9(2)	-8.9(2)	-0.33(19)
Si1	28.0(4)	34.0(4)	38.4(5)	-5.2(3)	-8.0(3)	0.7(3)
Mg1	32.4(5)	38.5(6)	49.4(7)	-12.4(5)	-12.7(5)	1.7(4)
O4	35.4(12)	48.7(13)	59.9(16)	-22.1(12)	-20.7(11)	6.0(10)
O2	33.7(12)	59.2(15)	50.3(15)	-3.5(12)	-13.5(11)	2.7(11)
O3	54.0(15)	43.0(13)	64.4(17)	-11.1(12)	-31.5(14)	-1.1(11)
O1	50.7(15)	45.6(13)	49.4(15)	-12.4(11)	-5.6(12)	7.0(11)
O5	46.7(14)	47.2(14)	55.3(16)	-17.8(12)	-7.6(12)	-4.8(11)
C9	33.7(16)	35.2(16)	44.6(19)	-5.9(14)	-8.2(14)	4.8(13)
C47	33.2(16)	36.2(16)	38.8(17)	-7.0(13)	-11.7(13)	-5.2(13)
C22	34.1(17)	53(2)	45(2)	-11.8(16)	-6.5(15)	0.5(15)
C48	41.1(18)	51(2)	41.5(19)	-6.3(16)	-15.2(15)	1.9(15)
C35	31.0(16)	43.4(18)	38.9(18)	0.8(14)	-4.9(13)	1.3(13)
C10	37.4(17)	39.8(17)	48(2)	-4.1(15)	-15.7(15)	2.8(14)
C1	34.6(17)	32.9(16)	70(3)	-9.2(17)	-13.5(17)	-4.5(13)
C42	35.0(17)	49.5(19)	46(2)	-7.1(16)	-12.2(15)	-5.8(15)
C41	34.5(16)	38.4(17)	45.2(19)	-5.4(14)	-14.7(15)	-1.6(13)
C51	40.3(18)	43.4(18)	42.0(19)	-10.4(15)	-5.5(15)	-0.8(15)
C46	40.7(18)	42.9(18)	48(2)	-10.2(16)	-10.3(16)	-1.1(15)
C50	43.4(19)	54(2)	37.8(18)	-8.0(16)	-4.7(15)	-13.4(16)
C49	52(2)	49(2)	45(2)	-0.4(17)	-16.2(17)	-3.8(17)
C52	40.7(18)	39.9(17)	40.7(19)	-6.0(15)	-8.2(15)	2.6(14)
C11	44.0(19)	41.4(18)	54(2)	0.0(16)	-12.8(16)	2.7(15)

Atom	U*11*	U*22*	U*33*	U*23*	U*13*	U*12*
C23	40.6(18)	47.5(19)	36.8(18)	-0.5(15)	-5.5(15)	1.2(15)
C2	38.6(18)	44.7(18)	48(2)	-8.7(16)	-13.4(15)	-6.5(15)
C36	55(2)	38.5(18)	58(2)	-3.9(17)	-23.3(19)	7.4(16)
C40	40.0(19)	49(2)	67(3)	-4.5(19)	-17.7(18)	6.7(16)
C38	54(2)	55(2)	64(3)	8(2)	-5(2)	23(2)
C45	58(2)	45(2)	61(3)	-17.8(19)	-20(2)	-4.4(18)
C43	43(2)	56(2)	54(2)	-1.3(18)	-15.9(17)	-17.0(17)
C53	41.6(19)	60(2)	72(3)	-31(2)	-17.8(19)	8.3(17)
C3	36.1(17)	45.6(19)	53(2)	-7.0(16)	-14.1(16)	-6.2(15)
C39	43(2)	68(3)	76(3)	0(2)	-21(2)	12.8(19)
C4	38.4(19)	60(2)	58(2)	-3.8(19)	-10.6(17)	-7.0(17)
C12	52(8)	43(11)	73(8)	10(7)	-19(6)	-10(8)
C81	49(2)	59(2)	53(2)	-15.9(19)	-14.2(18)	8.6(18)
C75	63(13)	54(7)	62(10)	-19(7)	-37(10)	9(6)
C64	39.7(19)	69(2)	59(2)	-9(2)	-16.6(18)	13.9(17)
C16	51(7)	36(10)	56(6)	-6(5)	-11(5)	5(5)
C5	39(2)	72(3)	69(3)	-13(2)	-2(2)	-8.8(19)
C37	67(3)	42(2)	58(3)	-3.9(18)	-14(2)	12.6(19)
C7	48(2)	58(2)	107(4)	-13(3)	-42(3)	4.5(19)
C8	50(2)	56(2)	63(3)	-5(2)	-25(2)	-4.9(18)
C44	71(14)	52(8)	50(13)	-9(7)	-8(9)	5(8)
C54	56(2)	50(2)	64(3)	-9.2(19)	-22(2)	-13.8(18)
C55	60(2)	48(2)	78(3)	-24(2)	-29(2)	9.7(18)
C6	54(2)	60(2)	72(3)	-23(2)	-26(2)	0.6(19)
C71	36(2)	72(3)	98(4)	-22(3)	-15(2)	3(2)
C15	71(3)	51(2)	71(3)	-12(2)	-42(2)	2.0(19)

Atom	U11	U22	U33	U23	U13	U12
C56	80(14)	60(12)	34(10)	-13(10)	-12(9)	-2(10)
C80	80(9)	68(10)	45(7)	7(7)	-25(6)	-24(6)
C57	47(2)	77(3)	103(4)	-45(3)	-40(2)	10(2)
C65	63(3)	98(4)	60(3)	-29(3)	-21(2)	-1(3)
C58	83(3)	69(2)	51(2)	-13.1(19)	-20(2)	16(2)
C77	37(4)	106(9)	75(8)	-17(6)	-21(4)	5(5)
C78	82(5)	63(4)	59(4)	1(4)	-19(4)	25(4)
C76	73(13)	39(5)	66(8)	-3(5)	-40(9)	-11(6)
C13	68(3)	95(4)	88(4)	-49(3)	0(3)	-35(3)
C60	58(10)	40(5)	59(7)	-13(5)	-30(7)	2(5)
C67	50(6)	61(7)	49(9)	9(7)	-18(6)	-10(5)
C59	76(5)	51(4)	61(5)	-8(4)	-23(5)	19(4)
C79	41(4)	73(6)	71(6)	5(5)	-8(4)	-2(4)
C14	82(15)	61(13)	48(11)	-6(9)	-19(9)	-7(10)
C66	78(5)	57(4)	62(4)	-2(3)	-22(4)	20(4)
C63	67(3)	84(3)	106(4)	-45(3)	-34(3)	2(3)
C62	87(13)	54(9)	55(12)	-8(9)	-22(10)	0(10)
C61	86(14)	70(13)	51(12)	-12(12)	-20(10)	5(12)
C68	62(7)	76(8)	47(9)	6(7)	-20(6)	-21(6)
C69	44(4)	96(7)	90(7)	22(6)	-8(5)	-7(4)
C70	73(4)	46(2)	60(3)	-7(2)	-27(2)	12(2)
C74	83(4)	58(2)	63(3)	0(2)	-21(3)	24(3)
C73	79(4)	64(3)	58(3)	3(2)	-22(3)	23(3)
C72	45(6)	85(10)	80(12)	-25(7)	-38(7)	11(6)
C30	35(5)	72(8)	95(11)	-11(7)	-19(6)	-10(5)
C31	35(7)	90(12)	62(8)	6(7)	-12(5)	-7(7)

Atom	U*11*	U*22*	U*33*	U*23*	U*13*	U*12*
C32	55(7)	47(4)	79(6)	-21(4)	-36(5)	10(4)
C33	82(11)	52(5)	88(8)	-11(5)	-45(8)	11(5)
C34	52(7)	63(5)	60(6)	-11(5)	-22(5)	11(4)
C24	75(5)	54(4)	53(4)	-8(3)	-20(3)	3(3)
C29	98(7)	58(5)	59(4)	-15(4)	-28(4)	-4(5)
C28	91(6)	50(3)	62(5)	-16(3)	-9(4)	-1(3)
C27	80(5)	52(3)	68(5)	-8(3)	-17(3)	12(3)
C26	62(5)	54(3)	56(5)	-7(3)	-14(3)	11(3)
C25	54(2)	51(2)	43(2)	-7.9(17)	-4.7(17)	-1.6(17)
C18	73(6)	59(5)	43(7)	8(5)	-28(5)	-20(4)
C20	71(6)	58(6)	45(5)	4(4)	-30(4)	-16(4)
C19	96(8)	65(6)	54(6)	11(5)	-39(6)	-31(5)
C17	41(4)	31(5)	56(5)	-5(4)	-5(4)	4(3)
C21	64(6)	41(9)	57(5)	11(5)	-19(5)	-6(6)

Table 20. Hydrogen coordinates (x 10^4) and isotropic displacement parameters (Å^2 x 10^3) for 027akb24.

Atom	*x*	*y*	*z*	U(eq)
H	3694.1	9128.33	3063.53	47
HA	4873.81	9674.5	2936.95	47
HB	7349.02	7742.21	1691.12	54
HC	6875.32	8859.09	1704.47	54
HD	5842.56	6326.75	4565.02	53
HE	3948.3	9414.5	1814.86	50
HF	4967.82	6165.46	2278.72	56
HG	4128.08	6525.34	3072.1	56
HH	8126.62	8899.32	2486.37	53
HI	2444.07	7811.67	5283.47	52
HJ	5423.73	9408.86	4235.31	53
HK	2802.62	6539.91	6079.27	56
HL	4509.54	5791.69	5720.58	59
HM	3775.6	8345.19	4141.56	50
HN	6268.91	8593.69	701.95	53
HO	4092.75	7081.51	1592.17	53
HP	6372.01	5836.44	2776.79	60
HQ	8182.44	7567.37	3510.6	63
HR	9137.76	4819.38	3096.73	76
HS	6159.68	10934.29	4253.37	65
HT	8846.74	10429.7	2511.67	61
H53A	855.21	3974.24	4976.3	68
H53B	678.11	4562.78	4240.48	68
HU	9435.41	6314.25	3456.37	75
HV	2671.15	7317.69	3571.38	64
HW	6363.99	10211.94	2134.35	69

Atom	*x*	*y*	*z*	U(eq)
H81A	3500.49	1257.9	1969.14	65
H81B	3321.85	2295.78	1545.27	65
H75A	1569.5	4995.4	986.63	66
H75B	688.71	5198.84	1788.31	66
H64A	4486.62	1576.58	2877.63	67
H64B	4246.79	2124.71	3626.02	67
H64C	4519.6	1610.34	2827	67
H64D	4144.44	2047.04	3621.77	67
HX	4842.08	10425.01	557.07	59
HY	915.9	7914.88	4074.67	76
HZ	7625.67	4593.6	2740.45	70
H1	838.8	8701.15	1978.19	80
H2	2609.66	8143.02	1462.53	66
H3	7149.75	6166.52	1236.14	73
H54A	7872.83	11445.5	3399.71	67
H54B	1321.68	5511.62	5098.97	71
H55A	1846.87	5797.9	4196	71
H55B	3410.07	5424.27	4475.29	72
H4	2793.23	4589.51	5147.89	72
H71C	-5.57	8593.42	3285.6	84
H71D	856.37	3491.22	2071.44	71
H71A	2060.38	3581.85	1445.3	71
H71B	807.83	3565.48	2076.43	71
H5	2021.93	3525.78	1465.82	71
H56A	5359.26	7712.61	134.47	71
H56B	6206.8	11541.36	-188.79	77

Atom	*x*	*y*	*z*	U(eq)
H80A	3658.6	4434.24	3594.23	83
H80B	3487.79	3581.7	4281.88	83
H57A	2744.28	1486.46	798.41	86
H57B	2738.96	467.59	1309.01	86
H57C	1307.6	2305.06	4999.99	82
H57D	2398.77	1744.71	5040.4	82
H65A	1113.75	2140.85	5010.58	82
H65B	2358.93	2028.4	4988.85	82
H58A	5651.97	3234.16	3042.29	86
H58B	6172.52	2305.39	2610.85	86
H77A	1074.9	526.87	5659.64	84
H77B	351.61	850.48	5130.1	84
H78A	3002.82	5101.03	2510.38	66
H78B	1717.93	5326.56	2869.8	66
H76A	1055.1	618.22	2739.79	104
H76B	399.88	1610.14	2675.32	104
H6	3011.57	5457.16	1310.89	59
H60A	2095.31	6206.82	1723.49	59
H60B	7468.47	11581.45	1494.48	65
H67A	3261.78	705.39	4209.13	76
H67B	2833.79	490.92	3533.78	76
H59A	4309.83	4296.42	2440.83	79
H59B	4059.29	3752.84	1803.3	79
H79A	5081.14	6323.64	-367.11	77
H79B	2014.95	-481.37	4850.04	80
H7	1246.21	-199.76	4342.78	80

Atom	*x*	*y*	*z*	U(eq)
H66A	1005.93	1760.28	1424.85	98
H66B	944.92	587.79	1601.85	98
H63A	7012.89	4858.98	658.69	79
H63B	6196.76	4961.06	-260.4	83
H62A	7485.89	12152.76	266.95	74
H62B	5617.51	2842.14	1590.03	99
H61A	6050.47	3834.06	1783.89	99
H61B	2607.97	486.13	3480.78	70
H68A	1299.72	523.54	3890.62	70
H68B	2901.04	32.17	4603.08	84
H69A	1778.98	-560.24	4736.66	84
H69B	716.72	590.07	5424.54	82
H70A	1779.35	629.12	5681.68	82
H70B	6157.84	2178.65	2644.69	77
H74A	5705.19	2837.81	3335.57	77
H74B	6223.05	3534.75	1878.3	81
H73A	5522.74	4117.23	2569.87	81
H73B	4280.82	4012.75	1982.86	78
H72A	4710.21	2968.94	1710.78	78
H72B	2562.11	5251.8	2700.44	67
H8	1245.73	5169.07	2973.6	67
H9	2709.84	5637.8	1462.87	84
H10	1626.03	6217.11	1888.95	84
H11	1493.6	4993.2	1005.33	69
H12	484.81	5051.01	1782.94	69
H13	5517.78	7381.87	91.8	73

**Atom**	***x***	***y***	***z***	**U(eq)**
H14	5573.56	5888.64	-353.82	85
H15	6535.09	4606.55	73.41	85
H16	7403.13	4872.15	919.01	83
H17	7331.86	6359.1	1373.91	71
H18	7479.22	11673.71	1170.2	68
H19	5940.58	11240.57	-309.79	67
H20	7263.77	12074.92	2.6	83
H21	6242.67	10612.6	2130.38	54
H22	4872.39	10020.81	569.39	67

Table 21. Torsion angles [°] for 027akb24.

A	B	C	D	Angle/°	A	B	C	D	Angle/°
Fe1	C9	C10	C11	-106.4(3)	C52	C47	C48	C49	0.0(5)
Fe1	C22	C23	C24	-105.4(3)	C52	C51	C50	C49	-0.2(6)
Fe1	C10	C11	C12	-60.8(19)	C11	C12	C13	C14	-10(5)
Fe1	C10	C11	C16	120.7(18)	C11	C16	C15	C14	-5(4)
Fe1	C10	C11	C17	-71.3(13)	C23	C24	C29	C28	-178.6(4)
Fe1	C10	C11	C21	109.5(14)	C23	C24	C25	C26	177.9(4)
Fe1	C1	C2	C3	-106.2(3)	C2	C3	C4	C5	-0.8(6)
Fe1	C23	C24	C30	-72(4)	C2	C3	C8	C7	1.2(6)
Fe1	C23	C24	C34	106(2)	C36	C35	C40	C39	171.7(3)
Fe1	C23	C24	C29	108.7(7)	C40	C35	C36	C37	-0.8(6)
Fe1	C23	C24	C25	-74.0(7)	C43	C42	C41	Si1	-0.7(5)
Fe1	C2	C3	C4	-70.6(4)	C43	C42	C41	C46	34.0(5)
Fe1	C2	C3	C8	110.4(4)	C53	O4	C56	C55	0.5(7)
Si1	C47	C48	C49	-176.7(3)	C53	C54	C55	C56	-1.1(7)
Si1	C47	C52	C51	176.5(3)	C3	C4	C5	C6	-1.3(6)
Si1	C35	C36	C37	-176.1(3)	C39	C38	C37	C36	-0.6(7)
Si1	C35	C40	C39	176.4(3)	C4	C3	C8	C7	1(4)
Si1	C41	C46	C45	-171.8(3)	C4	C5	C6	C7	6(4)
Mg1	O4	C53	C54	-156.0(3)	C12	C11	C16	C15	-28.0(6)
Mg1	O4	C56	C55	177.9(3)	C12	C13	C14	C15	-25.6(6)
Mg1	O2	C64	C65	-165.2(10)	C81	O5	C78	C79	14.7(17)
Mg1	O2	C64	C68	-157.2(18)	C81	C80	C79	C78	-39(2)
Mg1	O2	C67	C66	-169.0(11)	C64	O2	C67	C66	42(2)
Mg1	O2	C70	C69	152.7(11)	C64	O2	C70	C69	-12(3)
Mg1	O3	C71	C75	165(2)	C64	C65	C66	C67	7(4)

A	B	C	D	Angle/°	A	B	C	D	Angle/°
Mg1	O3	C71	C72	158.8(13)	C64	C68	C69	C70	2(4)
Mg1	O3	C77	C76	173.4(9)	C16	C11	C12	C13	1.4(7)
Mg1	O3	C74	C73	-178.7(9)	C16	C15	C14	C13	0.4(6)
Mg1	O1	C57	C58	132.1(11)	C37	C38	C39	C40	-0.2(7)
Mg1	O1	C57	C61	154.9(4)	C8	C3	C4	C5	-8(7)
Mg1	O1	C60	C59	-132.6(16)	C8	C7	C6	C5	-21.1(6)
Mg1	O1	C63	C62	-178.0(4)	C54	C55	C56	O4	1.2(7)
Mg1	O5	C81	C80	-163.8(3)	C6	C7	C8	C3	-17(2)
Mg1	O5	C78	C79	147.3(4)	C71	O3	C77	C76	-27.2(10)
O4	C53	C54	C55	-34.7(5)	C71	O3	C74	C73	-36(3)
O2	C64	C65	C66	-32.9(17)	C71	C75	C76	C77	11(6)
O2	C64	C68	C69	-13(3)	C56	O4	C53	C54	22.5(5)
O2	C67	C66	C65	-34(2)	C57	O1	C60	C59	54(2)
O3	C71	C72	C73	18(2)	C57	O1	C63	C62	-26.2(7)
O3	C77	C76	C75	33(3)	C57	C58	C59	C60	-3(3)
O3	C74	C73	C72	38.7(15)	C77	O3	C71	C75	-5(3)
O1	C57	C58	C59	35(2)	C78	O5	C81	C80	11.5(5)
O1	C57	C61	C62	20.1(7)	C76	C75	C71	O3	25(3)
O1	C60	C59	C58	-30(3)	C60	O1	C57	C58	-53.1(17)
O1	C63	C62	C61	37.9(7)	C67	O2	C64	C65	11.0(14)
O5	C81	C80	C79	8.6(6)	C63	O1	C57	C61	-178(3)
O5	C78	C79	C80	33.0(6)	C63	C62	C61	C57	0(6)
C9	C10	C11	C12	17(2)	C68	C69	C70	O2	-179(3)
C9	C10	C11	C16	-161.5(18)	C70	O2	C64	C68	4(7)
C9	C10	C11	C17	6.5(13)	C74	O3	C71	C72	3.7(7)
C9	C10	C11	C21	-172.7(14)	C74	C73	C72	C71	-35.5(7)

A	B	C	D	Angle/°	A	B	C	D	Angle/°
C47	C48	C49	C50	0.1(6)	C30	C31	C32	C33	34(3)
C22	C23	C24	C30	5(4)	C31	C30	C24	C23	33(2)
C22	C23	C24	C34	-177(2)	C31	C30	C24	C34	5.5(15)
C22	C23	C24	C29	-174.2(7)	C31	C32	C33	C34	-34(2)
C22	C23	C24	C25	3.1(8)	C32	C33	C34	C24	-0.3(13)
C48	C47	C52	C51	-0.3(5)	C33	C34	C24	C23	179.1(8)
C35	C36	C37	C38	-0.3(7)	C33	C34	C24	C30	1.7(16)
C35	C40	C39	C38	-0.4(7)	C24	C30	C31	C32	0.7(13)
C10	C11	C12	C13	-172(3)	C24	C29	C28	C27	0.2(15)
C10	C11	C16	C15	179.5(18)	C29	C24	C25	C26	-178.8(8)
C10	C11	C17	C18	179.0(12)	C29	C28	C27	C26	-1.3(15)
C10	C11	C21	C20	176.2(17)	C28	C27	C26	C25	0(8)
C1	C2	C3	C4	7.6(6)	C27	C26	C25	C24	-7(6)
C1	C2	C3	C8	-171.5(3)	C25	C24	C29	C28	-0.9(16)
C42	C41	C46	C45	0.7(6)	C19	C18	C17	C11	6(3)
C42	C43	C44	C45	-0.4(7)	C19	C20	C21	C11	4(3)
C41	C42	C43	C44	0.7(6)	C17	C11	C21	C20	-3(3)
C41	C46	C45	C44	-0.5(7)	C17	C18	C19	C20	-5(3)
C51	C50	C49	C48	0.0(6)	C21	C11	C17	C18	-2(2)
C46	C45	C44	C43	0.3(7)	C21	C20	C19	C18	0(2)
C50	C51	C52	C47	0.4(6)					

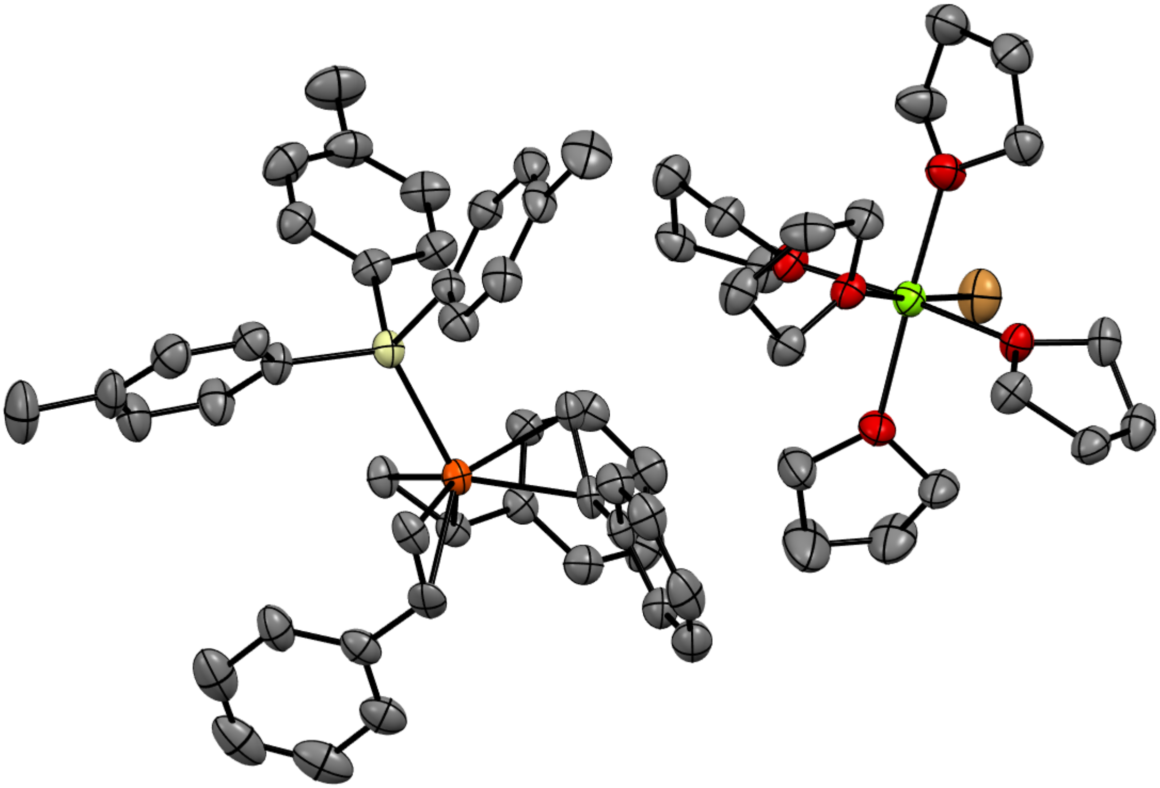

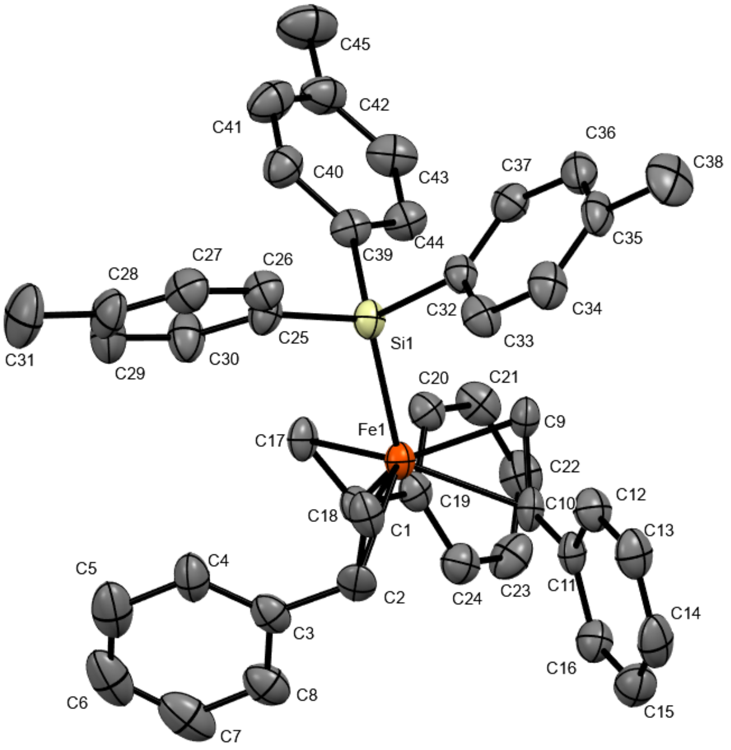
C45
C42
C41
C40
C43
C36
C38
C37
C35
C44
C39
C27
C26
C28
C32
C34
C25
Si1
C31
C29
C30
C33
C20
C21
C9
Fe1
C17
C22
C12
C18
C19
C10
C13
C1
C5
C4
C2
C24
C23
C11
C14
C3
C16
C8
C6
C7
C15

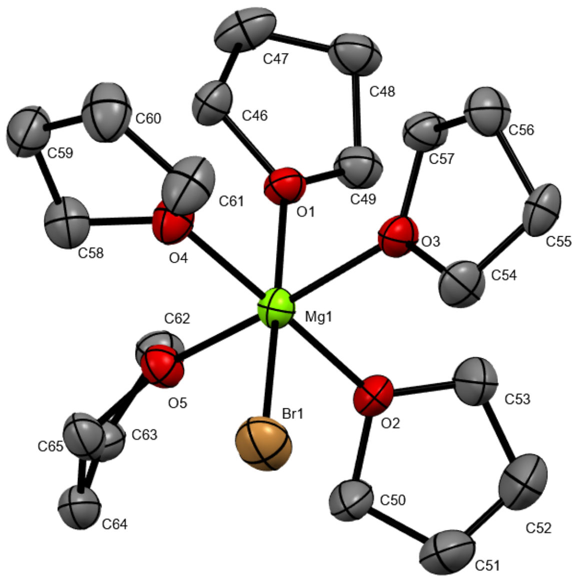

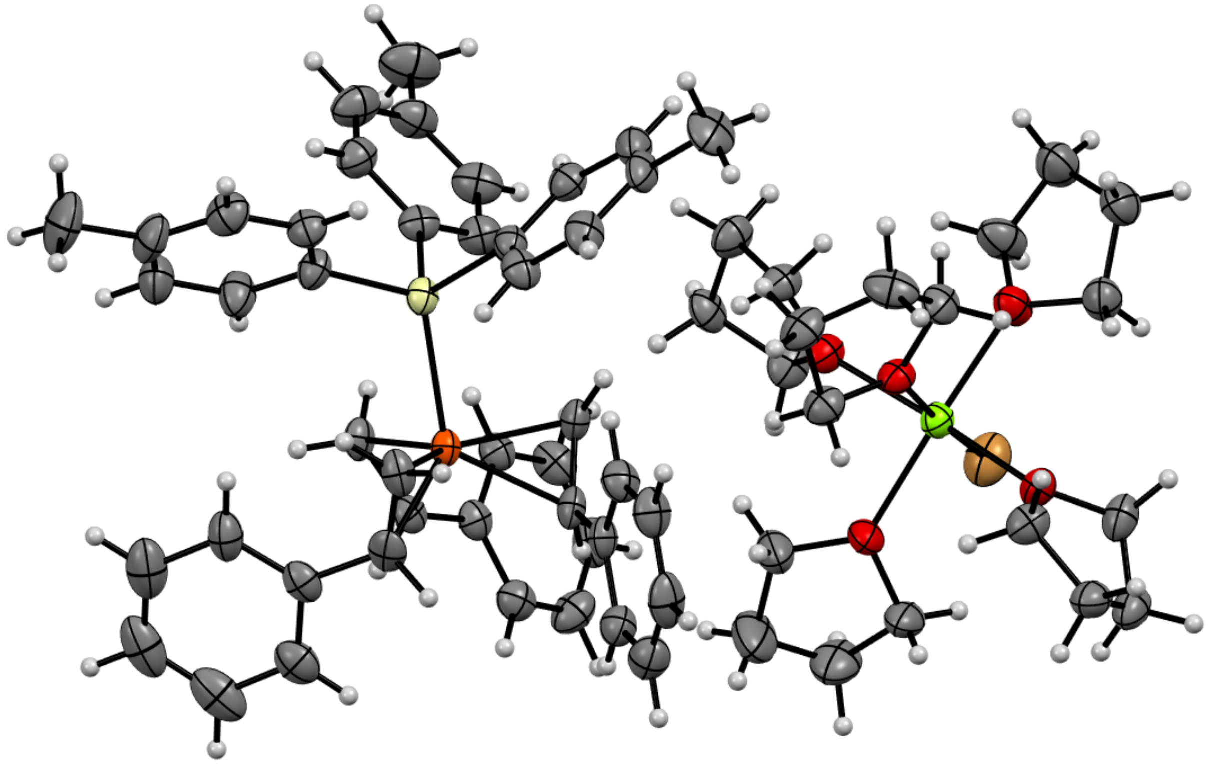

Reference Number: 098akb25

CCDC Number: 2484415

Crystal Structure Report: **Fe(styrene)$_3$Si(*p*-tolyl)$_3$**

Table 22. Crystal Data and structure refinement for **Fe(styrene)$_3$Si(*p*-tolyl)$_3$**.

Identification code	098akb25	
Empirical formula	C73 H101 Br Fe Mg O5 Si	
Formula weight	1278.69	
Temperature	149.9(4) K	
Wavelength	Cu Kα (λ = 1.54184)	
Crystal system	monoclinic	
Space group	Cc	
Unit cell dimensions	*a* = 21.5148(7) Å	α = 90°
	b = 14.6398(4) Å	β = 107.493(3)°
	c = 22.7027(7) Å	γ = 90°
Volume	6820(4) Å^3	
Z	4	
Density (calculated)	1.245 mg/m^3	
Absorption coefficient	3.117 mm^{-1}	
F(000)	2728	
Crystal color, morphology	orange, block	
Crystal size	0.2 x 0.1 x 0.05 mm^3	
Theta range for data collection	7.418 to 154.158°	
Index ranges	$-27 \leq h \leq 26$, $-18 \leq k \leq 18$, $-26 \leq l \leq 28$	
Reflections collected	38462	
Independent reflections	11368[*R*(int) = 0.074]	
Observed reflections	38462	
Completeness to theta = 25.242°	100.0%	
Absorption correction	Multi-scan	
Max. and min. transmission	0.856 and 0.722	
Refinement method	Full-matrix least-squares on F^2	
Data / restraints / parameters	12368 / 2 / 670	
Goodness-of-fit on *F*2	1.023	
Final *R* indices [*I*>2sigma(*I*)]	*R*1 = 0.0618, *wR*2 = 0.1606	
R indices (all data)	*R*1 = 0.0674, *wR*2 = 0.1660	
Largest diff. peak and hole	0.50 and -0.51 e.Å^{-3}	
Flack Parameter	0.005(6)	

Table 23. Atomic coordinates (x 10^4) and equivalent isotropic displacement parameters ($Å^2$ x 10^3) for 098akb25. U_{eq} is defined as one third of the trace of the orthogonalized U_{ij} tensor.

Atom	*x*	*y*	*z*	U(eq)
Br1	5874.3(4)	11937.3(5)	7130.2(4)	57.6(2)
Fe1	3786.9(4)	7148.6(5)	4718.7(4)	26.48(18)
Si1	4539.2(7)	5910.0(10)	5050.2(7)	29.2(3)
Mg1	6141.7(9)	11035.5(12)	6256.6(9)	31.1(4)
O3	5872(2)	9768(3)	6552(2)	35.8(9)
O1	6422(2)	10223(3)	5586(2)	32.8(8)
O5	6395(2)	12188(3)	5816(2)	35.4(8)
O2	5193(2)	11224(3)	5638(2)	38.2(9)
O4	7114(2)	10884(3)	6824(2)	39.8(9)
C9	4519(3)	8035(4)	5191(3)	32.4(11)
C39	4719(3)	5554(4)	5897(3)	33.5(11)
C40	4749(3)	4635(4)	6083(3)	42.0(13)
C19	3176(3)	8156(4)	5633(3)	31.4(11)
C44	4820(3)	6218(4)	6364(3)	39.9(13)
C25	4268(3)	4783(4)	4617(3)	34.8(12)
C57	6117(3)	8873(4)	6466(3)	37.7(12)
C37	5935(3)	6248(4)	5478(3)	37.2(12)
C16	3868(3)	9556(4)	3792(3)	38.8(12)
C56	5990(4)	8284(5)	6961(3)	43.9(14)
C55	5355(3)	8677(5)	7015(3)	40.7(13)
C18	3023(3)	7491(4)	5132(3)	30.7(10)
C17	3214(3)	6558(4)	5202(3)	32.9(11)
C28	3799(4)	3150(4)	3954(3)	49.3(17)
C10	4084(3)	8548(3)	4714(3)	31.1(11)
C3	2460(3)	6826(5)	3720(3)	36.9(12)
C11	4242(3)	8871(4)	4159(3)	31.2(11)

Atom	*x*	*y*	*z*	U(eq)
C20	3542(3)	7958(4)	6240(3)	33.7(11)
C54	5461(4)	9679(5)	6965(3)	43.0(14)
C36	6548(3)	6460(4)	5410(4)	43.9(14)
C34	6075(3)	6458(5)	4314(4)	43.3(14)
C21	3629(3)	8600(5)	6710(3)	40.4(13)
C35	6619(3)	6559(4)	4829(4)	41.5(14)
C2	3104(3)	7248(4)	3786(2)	37.4(12)
C1	3661(3)	6748(4)	3815(3)	35.7(12)
C27	4375(4)	3541(5)	3950(3)	46.4(15)
C4	2350(4)	5896(5)	3689(3)	45.5(15)
C24	2915(3)	9043(4)	5512(3)	38.8(12)
C42	4960(3)	5047(5)	7158(3)	46.8(15)
C49	6015(3)	9762(4)	5050(3)	40.5(13)
C23	2998(4)	9678(4)	5981(3)	45.7(15)
C32	5391(3)	6153(4)	4972(3)	31.2(10)
C43	4942(4)	5969(5)	6981(3)	48.0(15)
C48	6436(4)	8986(5)	4929(4)	46.6(15)
C26	4609(3)	4328(4)	4266(3)	37.5(12)
C58	7636(3)	11510(5)	6850(3)	44.3(14)
C22	3362(4)	9460(5)	6583(3)	45.6(15)
C46	7114(3)	10024(4)	5648(3)	40.9(13)
C62	6320(3)	12217(4)	5157(3)	39.3(13)
C8	1919(4)	7387(6)	3681(3)	46.8(15)
C15	4000(4)	9894(5)	3274(3)	46.1(14)
C12	4764(3)	8523(5)	3977(3)	39.3(13)
C64	6211(4)	13731(4)	5541(4)	46.6(15)
C29	3448(4)	3587(5)	4291(4)	50.0(16)
C33	5467(3)	6275(4)	4389(3)	37.9(12)

Atom	*x*	*y*	*z*	U(eq)
C14	4523(4)	9546(5)	3105(3)	49.5(17)
C50	4890(3)	12120(5)	5563(4)	50.6(17)
C30	3684(3)	4394(5)	4620(4)	47.2(16)
C65	6568(4)	13099(4)	6060(4)	44.0(14)
C13	4900(3)	8863(5)	3458(3)	43.3(14)
C60	8021(4)	10353(6)	7612(4)	56.1(18)
C63	6233(3)	13213(4)	4965(3)	43.2(14)
C41	4875(4)	4395(5)	6701(4)	50.1(16)
C5	1731(5)	5531(6)	3622(3)	59(2)
C45	5069(5)	4782(7)	7823(4)	67(2)
C59	8246(3)	11011(5)	7187(4)	49.9(16)
C7	1299(4)	7024(7)	3619(3)	61(2)
C38	7283(4)	6754(6)	4752(5)	60(2)
C53	4686(4)	10585(5)	5377(5)	56.6(19)
C6	1212(4)	6093(7)	3598(3)	61(2)
C31	3532(6)	2284(6)	3591(5)	71(3)
C61	7294(4)	10450(6)	7421(4)	54.3(18)
C47	7117(4)	9400(6)	5123(4)	52.2(17)
C52	4047(4)	11054(7)	5294(7)	86(4)
C51	4222(4)	11940(6)	5611(6)	66(2)

Table 24. Bond Lengths for 098akb25.

Atom	Atom	Length/Å	Atom	Atom	Length/Å
Br1	Mg1	2.587(2)	C18	C17	1.421(8)
Fe1	Si1	2.3962(17)	C28	C27	1.367(11)
Fe1	C9	2.073(5)	C28	C29	1.383(11)
Fe1	C18	2.182(5)	C28	C31	1.527(9)
Fe1	C17	2.069(5)	C10	C11	1.478(7)
Fe1	C10	2.148(5)	C3	C2	1.483(9)
Fe1	C2	2.190(5)	C3	C4	1.380(10)
Fe1	C1	2.072(6)	C3	C8	1.404(10)
Si1	C39	1.916(6)	C11	C12	1.403(8)
Si1	C25	1.920(6)	C20	C21	1.392(9)
Si1	C32	1.926(6)	C36	C35	1.379(11)
Mg1	O3	2.113(4)	C34	C35	1.391(11)
Mg1	O1	2.156(4)	C34	C33	1.395(9)
Mg1	O5	2.115(4)	C21	C22	1.378(10)
Mg1	O2	2.119(5)	C35	C38	1.518(9)
Mg1	O4	2.113(5)	C2	C1	1.390(10)
O3	C57	1.448(7)	C27	C26	1.372(9)
O3	C54	1.473(7)	C4	C5	1.400(11)
O1	C49	1.436(8)	C24	C23	1.384(10)
O1	C46	1.481(7)	C42	C43	1.405(11)
O5	C62	1.455(7)	C42	C41	1.381(12)
O5	C65	1.449(7)	C42	C45	1.508(10)
O2	C50	1.452(8)	C49	C48	1.528(9)
O2	C53	1.424(8)	C23	C22	1.393(11)
O4	C58	1.437(8)	C32	C33	1.392(8)
O4	C61	1.441(8)	C48	C47	1.523(12)
C9	C10	1.416(9)	C58	C59	1.497(10)
C39	C40	1.406(9)	C46	C47	1.503(10)

Atom	Atom	Length/Å	Atom	Atom	Length/Å
C39	C44	1.406(9)	C62	C63	1.517(9)
C40	C41	1.392(10)	C8	C7	1.403(11)
C19	C18	1.459(8)	C15	C14	1.390(11)
C19	C20	1.397(8)	C12	C13	1.389(9)
C19	C24	1.408(9)	C64	C65	1.513(11)
C44	C43	1.393(10)	C64	C63	1.525(10)
C25	C26	1.403(8)	C29	C30	1.408(10)
C25	C30	1.382(9)	C14	C13	1.382(12)
C57	C56	1.506(8)	C50	C51	1.498(11)
C37	C36	1.410(9)	C60	C59	1.541(11)
C37	C32	1.379(9)	C60	C61	1.500(11)
C16	C11	1.394(9)	C5	C6	1.374(15)
C16	C15	1.381(10)	C7	C6	1.374(14)
C56	C55	1.522(9)	C53	C52	1.497(12)
C55	C54	1.494(9)	C52	C51	1.476(14)

Table 25. Bond Angles for 098akb25.

Atom	Atom	Atom	Angle/˚	Atom	Atom	Atom	Angle/˚
C9	Fe1	Si1	88.55(17)	C57	C56	C55	102.2(5)
C9	Fe1	C18	100.8(2)	C54	C55	C56	101.7(5)
C9	Fe1	C10	39.1(2)	C19	C18	Fe1	117.7(4)
C9	Fe1	C2	130.0(2)	C17	C18	Fe1	66.3(3)
C18	Fe1	Si1	124.32(16)	C17	C18	C19	124.4(5)
C18	Fe1	C2	91.5(2)	C18	C17	Fe1	74.8(3)
C17	Fe1	Si1	88.33(18)	C27	C28	C29	117.2(6)
C17	Fe1	C9	117.9(2)	C27	C28	C31	122.6(8)
C17	Fe1	C18	39.0(2)	C29	C28	C31	120.2(8)
C17	Fe1	C10	128.8(2)	C9	C10	Fe1	67.6(3)
C17	Fe1	C2	101.9(2)	C9	C10	C11	123.7(5)
C17	Fe1	C1	119.0(2)	C11	C10	Fe1	117.0(4)
C10	Fe1	Si1	123.33(17)	C4	C3	C2	123.8(6)
C10	Fe1	C18	92.7(2)	C4	C3	C8	116.6(6)
C10	Fe1	C2	92.6(2)	C8	C3	C2	119.6(6)
C2	Fe1	Si1	123.22(19)	C16	C11	C10	120.0(5)
C1	Fe1	Si1	88.4(2)	C16	C11	C12	117.1(5)
C1	Fe1	C9	122.9(2)	C12	C11	C10	122.9(6)
C1	Fe1	C18	126.8(2)	C21	C20	C19	121.5(6)
C1	Fe1	C10	102.5(2)	O3	C54	C55	105.6(5)
C1	Fe1	C2	37.9(3)	C35	C36	C37	120.4(6)
C39	Si1	Fe1	115.74(17)	C35	C34	C33	120.1(6)
C39	Si1	C25	102.7(3)	C22	C21	C20	120.4(6)
C39	Si1	C32	103.4(3)	C36	C35	C34	119.0(6)
C25	Si1	Fe1	114.6(2)	C36	C35	C38	120.6(7)
C25	Si1	C32	105.2(2)	C34	C35	C38	120.4(7)
C32	Si1	Fe1	113.76(18)	C3	C2	Fe1	113.9(4)

Atom	Atom	Atom	Angle/°	Atom	Atom	Atom	Angle/°
O3	Mg1	Br1	93.38(13)	C1	C2	Fe1	66.5(3)
O3	Mg1	O1	84.35(17)	C1	C2	C3	123.5(6)
O3	Mg1	O5	170.2(2)	C2	C1	Fe1	75.6(3)
O3	Mg1	O2	91.33(19)	C28	C27	C26	122.6(7)
O1	Mg1	Br1	175.34(15)	C3	C4	C5	121.6(8)
O5	Mg1	Br1	95.96(13)	C23	C24	C19	121.2(6)
O5	Mg1	O1	86.54(17)	C43	C42	C45	121.1(8)
O5	Mg1	O2	85.17(19)	C41	C42	C43	117.6(6)
O2	Mg1	Br1	92.86(14)	C41	C42	C45	121.3(7)
O2	Mg1	O1	91.27(18)	O1	C49	C48	105.0(5)
O4	Mg1	Br1	89.63(15)	C24	C23	C22	120.4(6)
O4	Mg1	O3	91.72(18)	C37	C32	Si1	122.2(5)
O4	Mg1	O1	86.37(19)	C37	C32	C33	117.8(5)
O4	Mg1	O5	91.39(18)	C33	C32	Si1	119.9(4)
O4	Mg1	O2	175.9(2)	C44	C43	C42	121.3(7)
C57	O3	Mg1	127.3(3)	C47	C48	C49	102.9(6)
C57	O3	C54	108.6(4)	C27	C26	C25	121.6(6)
C54	O3	Mg1	123.6(4)	O4	C58	C59	105.3(5)
C49	O1	Mg1	129.0(4)	C21	C22	C23	119.3(6)
C49	O1	C46	109.1(4)	O1	C46	C47	106.7(5)
C46	O1	Mg1	121.9(4)	O5	C62	C63	106.8(5)
C62	O5	Mg1	123.0(4)	C7	C8	C3	121.9(8)
C65	O5	Mg1	128.8(4)	C16	C15	C14	119.7(7)
C65	O5	C62	107.6(5)	C13	C12	C11	120.6(6)
C50	O2	Mg1	120.3(4)	C65	C64	C63	102.9(5)
C53	O2	Mg1	130.9(4)	C28	C29	C30	120.8(7)
C53	O2	C50	106.5(5)	C32	C33	C34	121.6(6)
C58	O4	Mg1	125.2(4)	C13	C14	C15	119.1(6)

Atom	Atom	Atom	Angle/°	Atom	Atom	Atom	Angle/°
C58	O4	C61	104.9(5)	O2	C50	C51	103.8(6)
C61	O4	Mg1	123.8(4)	C25	C30	C29	121.7(6)
C10	C9	Fe1	73.3(3)	O5	C65	C64	104.7(6)
C40	C39	Si1	122.5(5)	C14	C13	C12	121.1(6)
C44	C39	Si1	120.5(4)	C61	C60	C59	104.9(6)
C44	C39	C40	116.9(6)	C62	C63	C64	105.4(6)
C41	C40	C39	121.4(7)	C42	C41	C40	121.6(7)
C20	C19	C18	124.0(5)	C6	C5	C4	120.7(8)
C20	C19	C24	117.2(5)	C58	C59	C60	103.4(6)
C24	C19	C18	118.7(5)	C6	C7	C8	119.7(8)
C43	C44	C39	121.1(6)	O2	C53	C52	108.2(6)
C26	C25	Si1	124.6(5)	C5	C6	C7	119.5(7)
C30	C25	Si1	119.3(5)	O4	C61	C60	106.0(6)
C30	C25	C26	116.0(6)	C46	C47	C48	103.8(5)
O3	C57	C56	105.1(5)	C51	C52	C53	104.5(8)
C32	C37	C36	121.1(6)	C52	C51	C50	103.3(7)
C15	C16	C11	122.4(6)				

Table 26. Anisotropic displacement parameters (Å2x 103) for 098akb25. The anisotropic displacement factor exponent takes the form: -2p2[h2 a*2U11 + ... + 2 h k a* b* U12]

Atom	U*11*	U*22*	U*33*	U*23*	U*13*	U*12*
Br1	71.0(5)	52.5(4)	58.3(5)	-8.5(3)	32.9(4)	-4.4(4)
Fe1	27.7(4)	27.9(3)	27.0(4)	-2.5(3)	13.1(3)	-4.5(3)
Si1	29.4(7)	29.6(6)	32.9(7)	-3.2(5)	15.9(6)	-3.2(5)
Mg1	29.2(9)	30.4(8)	35.6(9)	1.1(7)	12.8(7)	-1.4(7)
O3	34(2)	34(2)	47(2)	5.2(16)	23.6(18)	0.4(15)
O1	33(2)	30.8(18)	39(2)	0.4(15)	17.7(16)	3.5(15)
O5	41(2)	31.3(19)	36(2)	1.8(16)	14.2(17)	-1.7(16)
O2	24.8(19)	37(2)	52(3)	-1.0(18)	10.9(17)	0.7(15)
O4	34(2)	47(2)	38(2)	6.1(18)	10.8(17)	-3.5(18)
C9	37(3)	28(2)	33(3)	-3.7(19)	12(2)	-11(2)
C39	34(3)	38(3)	33(3)	1(2)	17(2)	5(2)
C40	42(3)	39(3)	49(4)	4(3)	20(3)	1(3)
C19	29(3)	39(3)	32(3)	0(2)	16(2)	-5(2)
C44	43(3)	41(3)	38(3)	2(2)	15(3)	4(2)
C25	41(3)	27(2)	40(3)	-5(2)	18(2)	-3(2)
C57	39(3)	35(3)	46(3)	4(2)	24(3)	5(2)
C37	41(3)	32(3)	40(3)	-1(2)	15(2)	2(2)
C16	41(3)	34(3)	43(3)	-3(2)	15(3)	-3(2)
C56	49(4)	39(3)	50(4)	8(3)	24(3)	-2(3)
C55	29(3)	54(4)	43(3)	4(3)	16(2)	-8(2)
C18	27(2)	37(3)	31(3)	1(2)	14(2)	-3(2)
C17	33(3)	35(3)	37(3)	-1(2)	21(2)	-10(2)
C28	69(5)	30(3)	46(4)	-9(3)	14(3)	-13(3)
C10	36(3)	25(2)	38(3)	-9(2)	19(2)	-10(2)
C3	34(3)	51(3)	25(2)	-2(2)	8(2)	-5(2)
C11	31(3)	33(2)	34(3)	-3(2)	16(2)	-12(2)

Atom	U*11*	U*22*	U*33*	U*23*	U*13*	U*12*
C20	31(3)	39(3)	34(3)	-2(2)	13(2)	-1(2)
C54	48(4)	46(3)	47(3)	-1(3)	32(3)	-2(3)
C36	34(3)	35(3)	62(4)	-2(3)	13(3)	-3(2)
C34	39(3)	43(3)	57(4)	-5(3)	28(3)	-2(2)
C21	37(3)	56(4)	32(3)	-6(3)	16(2)	0(3)
C35	27(3)	36(3)	66(4)	-2(3)	22(3)	-6(2)
C2	48(3)	44(3)	14(2)	1(2)	1(2)	-9(3)
C1	37(3)	47(3)	27(2)	-8(2)	15(2)	-14(2)
C27	55(4)	42(3)	43(3)	-6(3)	16(3)	2(3)
C4	48(4)	50(4)	41(3)	-3(3)	15(3)	-19(3)
C24	37(3)	44(3)	38(3)	3(2)	16(2)	-1(2)
C42	40(3)	62(4)	39(3)	16(3)	14(3)	4(3)
C49	44(3)	38(3)	42(3)	-2(2)	17(3)	3(2)
C23	57(4)	35(3)	54(4)	0(3)	30(3)	5(3)
C32	27(3)	29(2)	40(3)	-4(2)	14(2)	-1.1(19)
C43	47(4)	59(4)	40(3)	1(3)	17(3)	10(3)
C48	58(4)	37(3)	53(4)	-3(3)	30(3)	5(3)
C26	39(3)	36(3)	40(3)	-5(2)	15(2)	3(2)
C58	44(4)	44(3)	45(3)	0(3)	12(3)	-6(3)
C22	50(4)	50(4)	42(3)	-16(3)	21(3)	-5(3)
C46	31(3)	42(3)	55(4)	1(3)	21(3)	-1(2)
C62	47(3)	39(3)	39(3)	2(2)	22(3)	4(2)
C8	43(4)	63(4)	31(3)	-3(3)	7(3)	-1(3)
C15	55(4)	44(3)	38(3)	1(3)	11(3)	-5(3)
C12	34(3)	44(3)	44(3)	-2(3)	19(3)	-3(2)
C64	43(4)	33(3)	71(5)	0(3)	29(3)	-2(2)
C29	48(4)	43(3)	60(4)	-7(3)	19(3)	-15(3)
C33	32(3)	44(3)	42(3)	-7(2)	17(2)	0(2)

Atom	U*11*	U*22*	U*33*	U*23*	U*13*	U*12*
C14	57(4)	59(4)	38(3)	1(3)	23(3)	-26(3)
C50	31(3)	40(3)	80(5)	9(3)	16(3)	5(3)
C30	39(3)	41(3)	68(5)	-9(3)	26(3)	-6(3)
C65	50(4)	34(3)	55(4)	-6(3)	27(3)	-9(3)
C13	44(3)	52(4)	42(3)	-5(3)	25(3)	-13(3)
C60	49(4)	60(4)	52(4)	8(3)	5(3)	-7(3)
C63	37(3)	39(3)	55(4)	7(3)	15(3)	5(2)
C41	55(4)	49(4)	54(4)	17(3)	27(3)	6(3)
C5	70(5)	73(5)	34(3)	-3(3)	17(3)	-31(4)
C45	69(6)	88(6)	45(4)	22(4)	20(4)	13(5)
C59	36(3)	53(4)	61(4)	5(3)	15(3)	-3(3)
C7	42(4)	107(7)	33(3)	-6(4)	13(3)	-2(4)
C38	34(3)	70(5)	87(6)	-1(4)	35(4)	2(3)
C53	37(4)	44(4)	83(6)	-12(3)	8(3)	-3(3)
C6	50(4)	103(7)	31(3)	-8(4)	14(3)	-29(4)
C31	100(7)	43(4)	73(6)	-20(4)	31(5)	-26(4)
C61	42(4)	73(5)	46(4)	20(4)	11(3)	1(3)
C47	45(4)	70(5)	45(4)	1(3)	19(3)	19(3)
C52	37(4)	67(6)	153(12)	-8(6)	25(5)	-4(4)
C51	50(5)	54(4)	101(7)	2(4)	36(5)	13(3)

Table 27. Hydrogen coordinates (x 10^4) and isotropic displacement parameters ($Å^2$ x 10^3) for 098akb25.

Atom	*x*	*y*	*z*	U(eq)
H9A	4529.4	8191.25	5618.03	39
H9B	4956.64	7921.88	5147.4	39
H40	4682.76	4167.47	5779.82	50
H44	4804.47	6847.19	6257.44	48
H57A	6589.34	8899.44	6512.08	45
H57B	5885.49	8630.23	6050.61	45
H37	5894.81	6168.85	5880.96	45
H16	3508.23	9799.36	3901.56	47
H56A	6345.46	8340.79	7355.5	53
H56B	5940.08	7633.97	6835.76	53
H55A	4976.5	8459.96	6675.9	49
H55B	5289.86	8520.66	7416.39	49
H18	2587.13	7590.06	4820.96	37
H17A	2885.59	6111.17	4970.36	40
H17B	3444.97	6356.65	5627.92	40
H10	3808.84	8984.94	4865.18	37
H20	3734.78	7371.37	6334.4	40
H54A	5040.68	9998.64	6788.44	52
H54B	5684.02	9942.68	7375.57	52
H36	6914.84	6533.9	5764.98	53
H34	6117.83	6513.34	3911.42	52
H21	3875.4	8445.39	7120.1	48
H2	3083.87	7863.5	3589.81	45
H1A	3961.71	7026.17	3610.95	43
H1B	3595.2	6085.22	3730.88	43
H27	4622.95	3255.6	3718.42	56
H4	2703.09	5493.19	3714.09	55
H24	2678	9208.01	5101.35	47

Atom	*x*	*y*	*z*	U(eq)
H49A	5882.84	10182.09	4692.55	49
H49B	5618.99	9517.41	5129.75	49
H23	2806.36	10266.33	5891.43	55
H43	5014.52	6431.38	7287.51	58
H48A	6414.17	8441.37	5180.01	56
H48B	6299.83	8813.75	4486.73	56
H26	5012.35	4570.89	4248.01	45
H58A	7588.99	12073.14	7074.88	53
H58B	7640.11	11678.69	6429.16	53
H22	3424.3	9899.74	6903.82	55
H46A	7326.98	9723.88	6049.11	49
H46B	7351.82	10595.6	5625.89	49
H62A	5934.66	11857.22	4924.83	47
H62B	6710.56	11960.99	5073.46	47
H8	1975.34	8031.47	3698.17	56
H15	3735.45	10361.8	3035.09	55
H12	5026.85	8049.2	4211.71	47
H64A	6435.02	14328.19	5573.08	56
H64B	5756.11	13832.95	5541.95	56
H29	3042.82	3339.97	4299.87	60
H33	5096.55	6233.27	4034.56	45
H14	4620.39	9775.13	2751.37	59
H50A	4867.91	12385.13	5155.62	61
H50B	5134.3	12542.49	5891.67	61
H30	3434.56	4677.45	4850.62	57
H65A	7044.44	13194.79	6170.36	53
H65B	6425.88	13201.22	6430.88	53
H13	5257.09	8622.21	3344.4	52

Atom	*x*	*y*	*z*	U(eq)
H60A	8147.37	9716.46	7553.77	67
H60B	8215.61	10524.13	8050.8	67
H63A	5823	13301.55	4625.71	52
H63B	6602.06	13426.18	4825.6	52
H41	4902.08	3766.62	6811.17	60
H5	1668.29	4888.35	3592.3	70
H45A	5418.19	5156.92	8089.53	100
H45B	5191.88	4135.73	7878.72	100
H45C	4666.42	4879.61	7932.08	100
H59A	8583.1	11437.75	7430.46	60
H59B	8423.43	10671.05	6895.95	60
H7	941.49	7419.66	3591.43	73
H38A	7553.37	7077.98	5116.88	90
H38B	7230.55	7132.99	4384.22	90
H38C	7493.66	6176.85	4704.51	90
H53A	4726.03	10052.31	5654.23	68
H53B	4715.03	10366.71	4973.44	68
H6	796.73	5840.66	3566.8	74
H31A	3394	2420.34	3147.96	107
H31B	3158.15	2061.51	3711.58	107
H31C	3872.48	1814.91	3681.05	107
H61A	7083.61	9843.31	7395.61	65
H61B	7159.1	10829.21	7722.77	65
H47A	7453.68	8919.62	5260.98	63
H47B	7198.87	9745.73	4779.43	63
H52A	3812.97	11144.1	4850.13	103
H52B	3767.22	10693.69	5483.16	103
H51A	4227.52	11899.09	6047.39	79

**Atom**	***x***	***y***	***z***	**U(eq)**
H51B	3912.86	12424.15	5401.87	79

Table 28. Torsion angles [°] for 098akb25.

A	B	C	D	Angle/°	A	B	C	D	Angle/°
Fe1	C9	C10	C11	-108.4(5)	C20	C19	C18	Fe1	-77.6(6)
Fe1	C10	C11	C16	116.4(5)	C20	C19	C18	C17	1.3(9)
Fe1	C10	C11	C12	-64.1(7)	C20	C19	C24	C23	-2.3(9)
Si1	C39	C40	C41	-179.4(5)	C20	C21	C22	C23	0.4(10)
Si1	C39	C44	C43	179.1(5)	C54	O3	C57	C56	-14.3(7)
Si1	C25	C26	C27	-177.6(5)	C36	C37	C32	Si1	-177.6(4)
Si1	C25	C30	C29	177.5(6)	C36	C37	C32	C33	-0.2(8)
Si1	C32	C33	C34	179.6(5)	C35	C34	C33	C32	-2.7(10)
Mg1	O3	C57	C56	157.6(4)	C2	C3	C4	C5	-179.5(6)
Mg1	O3	C54	C55	176.0(4)	C2	C3	C8	C7	-180.0(6)
Mg1	O1	C49	C48	-155.1(4)	C27	C28	C29	C30	-0.8(12)
Mg1	O1	C46	C47	176.6(4)	C4	C3	C2	Fe1	-75.7(7)
Mg1	O5	C62	C63	153.5(4)	C4	C3	C2	C1	0.9(9)
Mg1	O5	C65	C64	-137.4(5)	C4	C3	C8	C7	0.4(10)
Mg1	O2	C50	C51	-132.2(6)	C4	C5	C6	C7	2.2(11)
Mg1	O2	C53	C52	147.3(8)	C24	C19	C18	Fe1	105.8(5)
Mg1	O4	C58	C59	-166.4(4)	C24	C19	C18	C17	-175.2(5)
Mg1	O4	C61	C60	170.9(5)	C24	C19	C20	C21	1.8(8)
O3	C57	C56	C55	34.1(7)	C24	C23	C22	C21	-1.0(11)
O1	C49	C48	C47	-34.2(7)	C49	O1	C46	C47	-0.7(7)
O1	C46	C47	C48	-20.7(7)	C49	C48	C47	C46	33.2(7)
O5	C62	C63	C64	-4.2(7)	C32	C37	C36	C35	-1.2(9)
O2	C50	C51	C52	-37.3(11)	C43	C42	C41	C40	-2.5(11)
O2	C53	C52	C51	-8.6(13)	C26	C25	C30	C29	0.0(11)
O4	C58	C59	C60	-28.6(8)	C58	O4	C61	C60	-35.5(8)
C9	C10	C11	C16	-163.7(5)	C46	O1	C49	C48	21.9(6)
C9	C10	C11	C12	15.8(8)	C62	O5	C65	C64	33.7(6)

A	B	C	D	Angle/°	A	B	C	D	Angle/°
C39	C40	C41	C42	1.5(11)	C8	C3	C2	Fe1	104.7(6)
C39	C44	C43	C42	-0.9(11)	C8	C3	C2	C1	-178.6(6)
C40	C39	C44	C43	-0.2(10)	C8	C3	C4	C5	0.1(10)
C19	C18	C17	Fe1	-108.4(5)	C8	C7	C6	C5	-1.7(11)
C19	C20	C21	C22	-0.9(9)	C15	C16	C11	C10	178.9(6)
C19	C24	C23	C22	2.0(10)	C15	C16	C11	C12	-0.7(9)
C44	C39	C40	C41	-0.1(10)	C15	C14	C13	C12	-0.1(10)
C57	O3	C54	C55	-11.7(7)	C29	C28	C27	C26	0.5(11)
C57	C56	C55	C54	-40.4(7)	C33	C34	C35	C36	1.2(10)
C37	C36	C35	C34	0.7(9)	C33	C34	C35	C38	179.7(6)
C37	C36	C35	C38	-177.8(6)	C50	O2	C53	C52	-15.0(10)
C37	C32	C33	C34	2.1(9)	C30	C25	C26	C27	-0.3(10)
C16	C11	C12	C13	0.9(9)	C65	O5	C62	C63	-18.2(7)
C16	C15	C14	C13	0.3(10)	C65	C64	C63	C62	23.7(7)
C56	C55	C54	O3	32.3(7)	C63	C64	C65	O5	-35.1(6)
C18	C19	C20	C21	-174.8(5)	C41	C42	C43	C44	2.2(11)
C18	C19	C24	C23	174.4(6)	C45	C42	C43	C44	-177.8(7)
C28	C27	C26	C25	0.0(11)	C45	C42	C41	C40	177.5(7)
C28	C29	C30	C25	0.5(13)	C59	C60	C61	O4	16.8(9)
C10	C11	C12	C13	-178.7(6)	C53	O2	C50	C51	32.4(9)
C3	C2	C1	Fe1	-104.0(5)	C53	C52	C51	C50	27.8(12)
C3	C4	C5	C6	-1.4(11)	C31	C28	C27	C26	178.8(8)
C3	C8	C7	C6	0.4(11)	C31	C28	C29	C30	-179.1(8)
C11	C16	C15	C14	0.1(10)	C61	O4	C58	C59	40.4(7)
C11	C12	C13	C14	-0.5(10)	C61	C60	C59	C58	7.1(9)

5. References

[50] Neate, P. G. N.; Greenhalgh, M. D.; Brennessel, W. W.; Thomas, S. P.; Neidig, M. L. TMEDA in Iron Catalyzed Hydromagnesiation: Formation of Iron(II)-Alkyl Species for Controlled Reduction to Alkene-Stabilized Iron(0) *Angew. Chem. Int. Ed.* **2020**, *59*, 17070–17076. DOI: 10.1002/anie.202006639

[51] Horino, Y.; Ishibashi, M.; Nakasai, K.; Korenaga, T. Palladium-catalyzed reaction of γ-silylated allyl acetates proceeding through 1,2-shift of a substituent on silicon. *Tetrahedron* **2020**, *76* (40), 131493. DOI: 10.106/j.tet.2020.131493

[52] Evans, D. F. The Determination of the Paramagnetic Susceptibility of Substances in Solution by Nuclear Magnetic Resonance. *The Journal of Chemical Society* **1959**. DOI: 10.1039/JR9590002003.

[53] Bain, G. A.; Berry, J. F. Diamagnetic Corrections and Pascal's Constants. *Journal of Chemical Education* **2008**, *85* (4). DOI: 10.1021/ed085p532.

[54] Helmich-Paris, B.; de Souza, B.; Neese, F.; Izsák, R. An improved chain of spheres for exchange algorithm. *The Journal of Chemical Physics* **2021**, *155* (10). DOI: 10.1063/5.0058766.

[55] Neese, F. Software update: The ORCA program system—Version 5.0. *Wiley Interdisciplinary Reviews: Computational Molecular Science* **2022**, *12* (5), e1606. DOI: 10.1002/wcms.1606.

[56] Neese, F. The ORCA program system. *Wiley Interdisciplinary Reviews: Computational Molecular Science* **2012**, *2* (1), 73–78. DOI: 10.1002/wcms.81.

[57] Meng, E. C.; Goddard, T. D.; Pettersen, E. F.; Couch, G. S.; Pearson, Z. J.; Morris, J. H.; Ferrin, T. E. UCSF ChimeraX: Tools for structure building and analysis. *Protein Science* **2023**, *32* (11). DOI: 10.1002/pro.4792.

[58] Pettersen, E. F.; Goddard, T. D.; Huang, C. C.; Meng, E. C.; Couch, G. S.; Croll, T. I.; Morris, J. H.; Ferrin, T. E. UCSF ChimeraX: Structure visualization for researchers, educators, and developers. *Protein Science* **2021**, *30* (1). DOI: 10.1002/pro.3943.

[59] Staroverov, V. N.; Scuseria, G. E.; Tao, J.; Perdew, J. P. Comparative assessment of a new nonempirical density functional: Molecules and hydrogen-bonded complexes. *The Journal of Chemical Physics* **2004**, *121* (22). DOI: 10.1063/1.1795692.

[60] Tao, J.; Perdew, J. P.; Staroverov, V. N.; Scuseria, G. E. Climbing the Density Functional Ladder: Nonempirical Meta--Generalized Gradient Approximation Designed for Molecules and Solids. *Physical Review Letters* **2003**, *91* (14). DOI: 10.1103/PhysRevLett.91.146401.

[61] Pollak, P.; Weigend, F. Segmented Contracted Error-Consistent Basis Sets of Double- and Triple-ζ Valence Quality for One- and Two-Component Relativistic All-Electron Calculations. *Journal of Chemical Theory and Computation* **2017**, *13* (8). DOI: 10.1021/acs.jctc.7b00593.

[62] Cheng, L.; Gauss, J. Analytic energy gradients for the spin-free exact two-component theory using an exact block diagonalization for the one-electron Dirac Hamiltonian. *The Journal of Chemical Physics* **2011**, *135* (8). DOI: 10.1063/1.3624397.
[63] Cheng, L.; Gauss, J. Analytic second derivatives for the spin-free exact two-component theory. *The Journal of Chemical Physics* **2011**, *135* (24). DOI: 10.1063/1.3667202.
[64] Franzke, Y. J.; Middendorf, N.; Weigend, F. Efficient implementation of one- and two-component analytical energy gradients in exact two-component theory. *The Journal of Chemical Physics* **2018**, *148* (10). DOI: 10.1063/1.5022153.
[65] Neese, F.; Wennmohs, F.; Hansen, A.; Becker, U. Efficient, approximate and parallel Hartree–Fock and hybrid DFT calculations. A 'chain-of-spheres' algorithm for the Hartree–Fock exchange. *Chemical Physics* **2009**, *356* (1-3). DOI: 10.1016/j.chemphys.2008.10.036.
[66] Grimme, S.; Antony, J.; Ehrlich, S.; Krieg, H. A consistent and accurate ab initio parametrization of density functional dispersion correction (DFT-D) for the 94 elements H-Pu. *The Journal of Chemical Physics* **2010**, *132* (15). DOI: 10.1063/1.3382344.
[67] Grimme, S.; Ehrlich, S.; Goerigk, L. Effect of the damping function in dispersion corrected density functional theory. *Journal of Computational Chemistry* **2011**, *32* (7). DOI: 10.1002/jcc.21759.
[68] Chai, J.-D.; Chai, J.-D.; Head-Gordon, M.; Head-Gordon, M. Long-range corrected hybrid density functionals with damped atom–atom dispersion corrections. *Physical Chemistry Chemical Physics* **2008**, *10* (44). DOI: 10.1039/B810189B.
[69] Neese, F. Prediction and interpretation of the 57Fe isomer shift in Mössbauer spectra by density functional theory. *Inorganica Chimica Acta* **2002**, *337*. DOI: 10.1016/S0020-1693(02)01031-9.
[70] Santra, G.; Santra, G.; Neese, F.; Neese, F.; Pantazis, D. A.; Pantazis, D. A. Extensive reference set and refined computational protocol for calculations of 57Fe Mössbauer parameters. *Physical Chemistry Chemical Physics* **2024**, *26* (35). DOI: 10.1039/D4CP00431K.
[71] Angeli, C.; Cimiraglia, R.; Evangelisti, S.; Leininger, T.; Malrieu, J.-P. Introduction of n-electron valence states for multireference perturbation theory. *The Journal of Chemical Physics* **2001**, *114* (23). DOI: 10.1063/1.1361246.
[72] Guo, Y.; Sivalingam, K.; Valeev, E. F.; Neese, F. SparseMaps—A systematic infrastructure for reduced-scaling electronic structure methods. III. Linear-scaling multireference domain-based pair natural orbital N-electron valence perturbation theory. *The Journal of Chemical Physics* **2016**, *144* (9). DOI: 10.1063/1.4942769.
[73] Ugandi, M.; Roemelt, M. A recursive formulation of one-electron coupling coefficients for spin-adapted configuration interaction calculations featuring many unpaired electrons. *International Journal of Quantum Chemistry* **2023**, *123* (5). DOI: 10.1002/qua.27045.

[74] Kollmar, C.; Sivalingam, K.; Helmich-Paris, B.; Angeli, C.; Neese, F. A perturbation-based super-CI approach for the orbital optimization of a CASSCF wave function. *Journal of Computational Chemistry* **2019**, *40* (14). DOI: 10.1002/jcc.25801.
[75] Weigend, F.; Ahlrichs, R. Balanced basis sets of split valence, triple zeta valence and quadruple zeta valence quality for H to Rn: Design and assessment of accuracy. *Physical Chemistry Chemical Physics* **2005**, *7* (18), 3297–3305. DOI: 10.1039/B508541A.
[76] Hellweg, A.; Hättig, C.; Höfener, S.; Klopper, W.; Hellweg, A.; Hättig, C.; Höfener, S.; Klopper, W. Optimized accurate auxiliary basis sets for RI-MP2 and RI-CC2 calculations for the atoms Rb to Rn. *Theoretical Chemistry Accounts 2007 117:4* **2007**, *117* (4). DOI: 10.1007/s00214-007-0250-5.
[77] Agilent (**2014**). CrysAlis PRO. Agilent Technologies Ltd, Yarnton, Oxfordshire, England.
[78] Sheldrick, G.M. (**2015**). Acta Cryst. A71, 3-8.
[79] Sheldrick, G.M. (**2015**). Acta Cryst. C71, 3-8
[80] Dolomanov, O.V., Bourhis, L.J., Gildea, R.J, Howard, J.A.K. & Puschmann, H. (**2009**), J. Appl. Cryst. 42, 339-341.
